# HIV-1 Tropism Testing in Subjects Achieving Undetectable HIV-1 RNA: Diagnostic Accuracy, Viral Evolution and Compartmentalization

**DOI:** 10.1371/journal.pone.0067085

**Published:** 2013-08-01

**Authors:** Christian Pou, Francisco M. Codoñer, Alexander Thielen, Rocío Bellido, Susana Pérez-Álvarez, Cecilia Cabrera, Judith Dalmau, Marta Curriu, Yolanda Lie, Marc Noguera-Julian, Jordi Puig, Javier Martínez-Picado, Julià Blanco, Eoin Coakley, Martin Däumer, Bonaventura Clotet, Roger Paredes

**Affiliations:** 1 Institut de Recerca de la SIDA irsiCaixa – HIVACAT, Hospital Universitari Germans Trias i Pujol, Universitat Autònoma de Barcelona, Catalonia, Spain; 2 HIV Unit-Fundació Lluita contra la SIDA, Hospital Universitari Germans Trias i Pujol, Universitat Autònoma de Barcelona, Catalonia, Spain; 3 Max-Planck-Institut für Informatik, Saarbücken, Germany; 4 Institució Catalana de Recerca i Estudis Avançats (ICREA), Barcelona, Spain; 5 Monogram Biosciences Inc., South San Francisco, California, United States of America; 6 Institut für Immunologie und Genetik, Kaiserlautern, Germany; Rush University, United States of America

## Abstract

**Background:**

Technically, HIV-1 tropism can be evaluated in plasma or peripheral blood mononuclear cells (PBMCs). However, only tropism testing of plasma HIV-1 has been validated as a tool to predict virological response to CCR5 antagonists in clinical trials. The preferable tropism testing strategy in subjects with undetectable HIV-1 viremia, in whom plasma tropism testing is not feasible, remains uncertain.

**Methods & Results:**

We designed a proof-of-concept study including 30 chronically HIV-1-infected individuals who achieved HIV-1 RNA <50 copies/mL during at least 2 years after first-line ART initiation. First, we determined the diagnostic accuracy of 454 and population sequencing of gp120 V3-loops in plasma and PBMCs, as well as of MT-2 assays before ART initiation. The Enhanced Sensitivity Trofile Assay (ESTA) was used as the technical reference standard. 454 sequencing of plasma viruses provided the highest agreement with ESTA. The accuracy of 454 sequencing decreased in PBMCs due to reduced specificity. Population sequencing in plasma and PBMCs was slightly less accurate than plasma 454 sequencing, being less sensitive but more specific. MT-2 assays had low sensitivity but 100% specificity. Then, we used optimized 454 sequence data to investigate viral evolution in PBMCs during viremia suppression and only found evolution of R5 viruses in one subject. No *de novo* CXCR4-using HIV-1 production was observed over time. Finally, Slatkin-Maddison tests suggested that plasma and cell-associated V3 forms were sometimes compartmentalized.

**Conclusions:**

The absence of tropism shifts during viremia suppression suggests that, when available, testing of stored plasma samples is generally safe and informative, provided that HIV-1 suppression is maintained. Tropism testing in PBMCs may not necessarily produce equivalent biological results to plasma, because the structure of viral populations and the diagnostic performance of tropism assays may sometimes vary between compartments. Thereby, proviral DNA tropism testing should be specifically validated in clinical trials before it can be applied to routine clinical decision-making.

## Introduction

The efficacy of CCR5 antagonist therapy depends on the accurate characterization of HIV-1 tropism. A major cause of virological failure to CCR5-antagonist therapy is the emergence of pre-existing CXCR4-using viruses, often missed by tropism assays [1], [2]. Retrospective reanalyses of pre-treatment plasma samples from maraviroc trials in treatment-naïve [3] and -experienced [4], [5], [6] individuals found that population and 454 sequencing (454 Life Sciences/Roche) of the V3 loop of gp120 were able to predict virological response to maraviroc as accurately as Trofile™ and the enhanced sensitivity version of Trofile™ (ESTA), respectively [7], [8]. In the MERIT trial, first-line therapy with maraviroc in treatment naïve individuals only achieved non-inferiority to efavirenz when the ESTA [3] or 454 sequencing [9] and not the former, less sensitive version of Trofile™ were used to assess presence of CXCR4-using virus at screening. Moreover, the risk of virological failure to maraviroc-including therapy was directly proportional to the amount of CXCR4-using viruses in the viral population in other studies [10]. Presence of as little as 2% CXCR4-using viruses in the population conferred an increased risk of virological failure to maraviroc-including therapy. As with other regimens, lower CD4+ counts, resistance to other drugs or inclusion of less than 3 active drugs in the regimen further increased the risk of virological failure to maraviroc regimens.

Tropism assays validated in clinical trials to date characterize plasma viruses from subjects with detectable HIV-1 RNA. Maraviroc clinical trials enrolled individuals with HIV-1 RNA ≥2000 copies/mL (MERIT Study in ART-naïve individuals) and ≥5000 copies/mL (MOTIVATE 1&2 studies in treatment –experienced subjects). However, most HIV-1-infected individuals who could potentially benefit from the favorable toxicity and drug-drug-interaction profile of CCR5 antagonists have undetectable HIV-1 RNA levels under antiretroviral therapy (ART). The optimal strategy to evaluate HIV tropism in aviremic subjects, in whom plasma tropism testing is not feasible, remains uncertain. Testing of stored pre-therapy plasma samples, when available, may not capture potential virus evolution towards CXCR4-use during ART. Conversely, proviral DNA testing might assess different virus populations than those circulating simultaneously in plasma, implying that both tests may not necessarily provide equivalent genotypic or phenotypic information for clinical decision-making and may need to be validated independently in clinical trials.

We designed this study to gain further insight into the aforementioned questions. First, we investigated the ability of different state-of-the-art genotypic and phenotypic tropism tests in proviral DNA and plasma RNA to detect CXCR4-using viruses relative to the ESTA in 30 subjects before they initiated first-line ART without CCR5 antagonists. Then, we examined if virus evolution occurred after at least 2 years of persistent HIV-1 RNA suppression under ART in these same individuals. Finally, we used population differentiation tests to investigate if pre-treatment PBMC V3 form populations were different from a) those simultaneously observed in plasma and b) those observed in PBMCs after at least 2 years of continuous HIV-1 RNA suppression.

## Materials and Methods

### Study design and participants

**Figure 1:**
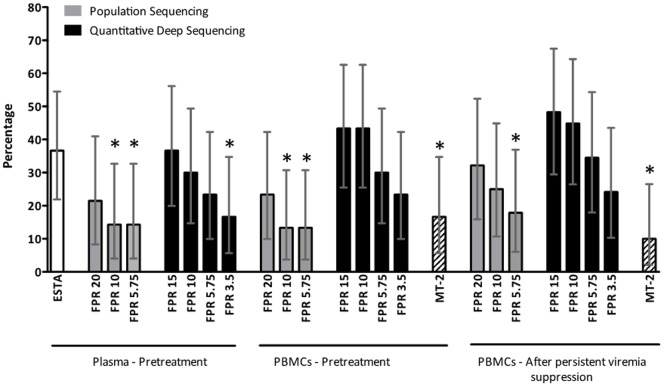
Prevalence of CXCR4-using viruses using different tropism assays and settings. Bar plot showing the mean and 95% confidence intervals of the prevalence of subjects with CXCR4-using viruses using different tropism assays and settings. The Geno2Pheno_[coreceptor]_ clinical model was only used in pre-treatment bulk sequences derived from plasma RNA; otherwise, the clonal model was used. ESTA, Enhanced-Sensitivity Trofile™ Assay; FPR, Geno2Pheno_[coreceptor]_ false positive rate used to assign tropism; MT-2, Direct cocultivation of patient-derived peripheral blood mononuclear cells with MT-2 cells. * p-value<0.05, two-sided exact binomial tst.

This was a retrospective proof-of-concept study that included chronically HIV-1-infected adults who achieved persistent HIV-1 RNA levels <50 copies/mL during at least 2 years after starting first-line ART without CCR5 antagonists. Subjects had to have cryopreserved samples available for testing within 6 months before ART initiation (baseline, T1) and after at least 2 years of undetectable viremia (persistent viremia suppression, T2). (Figure 1) The Institutional Review Board of the Hospital Universitari Germans Trias i Pujol, Badalona, Spain, approved the study; participants provided written informed consent for retrospective sample testing. Tropism tests performed at T1 included the *Enhanced-Sensitivity Trofile™Assay* (ESTA), direct cocultivation of patient-derived PBMCs with MT2 cells (MT2 assay), and population and 454 sequencing of the V3-loop in plasma RNA and proviral DNA. Tropism tests performed at T2 included population and 454 sequencing in PBMCs and the MT2 assay. HIV-1 RNA levels (NucliSens EasyQ HIV-1, Biomerieux, Marcy l'Etoile, France), CD4+ and CD8+ cells counts were determined at regular 3 to 4-month intervals as part of the routine clinical follow-up of subjects.

### Tropism testing

The ESTA was performed in Monogram Biosciences, South San Francisco, USA, blinded for clinical characteristics or other tropism testing results. ESTA was considered the technical reference standard for comparison with the remaining tropism assays because a) it has been used in most CCR5 antagonist clinical trials, b) is widely recognized as a sensitive, accurate and robust tropism test. c) is the only FDA-approved and CLIA-certified HIV tropism test and d) is readily available to HIV clinics worldwide. For the MT2 assay, HIV-1 was isolated by direct cocultivation with 1–5×10^6^ patient-derived cryopreserved PBMCs with 1×10^6^ MT2 cell line, in duplicate, as in [11]. Functional CXCR4-using viruses were defined by their ability to grow in MT2 cocultures, as confirmed by p24 production. Virus growth ability was evaluated with cocultures of 1×10^6^ patient-derived PHA-stimulated PBMCs and PHA-stimulated PBMC from healthy seronegative donors. For population and 454 V3 loop sequencing, HIV-1 RNA was extracted from 1 mL of plasma after ultracentrifugation at 30,000 rpm during 1.5 hours; HIV-1 DNA was extracted from 10 million PBMCs. Reverse transcription and DNA amplification were performed in triplicate parallel reactions followed by pooling of PCR products to avoid founder effects. First-round PCR products were used for both population and 454 V3-loop sequencing. The V3-loop of functional viruses extracted from the supernatant of positive MT2 assays was also sequenced for comparison with 454 sequencing. The combined error threshold for PCR amplification and 454 sequencing was established identifying the percentage of different V3-loop unique sequences (haplotypes) obtained after amplifying a commercial pNL4.3 DNA clone under the same PCR conditions used to generate patient samples and using the same filtering steps. A total of 2,702 V3-loop pNL4.3 clonal sequences and 154 different haplotypes were obtained by 454 sequencing, which followed a Poisson-like distribution. The 99^th^ percentile of such distribution established the threshold for detecting “valid” V3-loop haplotypes at ≥0.6% of the viral population. The percentage of valid V3-loop haplotypes with predicted CXCR4-using tropism was then calculated.

### Statistical analyses

Mean and 95% confidence intervals of the sensitivity, specificity, positive and negative predictive value and accuracy of each assay were calculated assuming a binomial distribution of the data. “Accuracy” (also known as “Fraction Correct”) was defined as: (True positives + True negatives)/Total. V3 loop genotypes derived from population and 454 sequencing were interpreted using Geno2Pheno_[coreceptor]_ [12] at false positive rates (FPR) ranging from 3.5% to 20%. The FPR cut-off providing better diagnostic performance relative to the ESTA was used to define viral tropism in subsequent analyses. HIV subtype was determined with the Geno2Pheno_[coreceptor]_ tool. The prevalence of subjects with CXCR4-using viruses was determined with each assay and setting; differences relative to ESTA were tested for significance using a two-sided exact binomial test. Statistical analyses were performed with *R* [13].

### Phylogenetic analyses

The phylogenetic relatedness between viruses present in plasma and PBMCs at T1 and in PBMCs at T2 was determined using valid 454 sequencing V3-loop haplotypes. Sequences were codon-aligned with HIValign; the optimal nucleotide evolution model was determined with FindModel (http://www.hiv.lanl.gov). Maximum likelihood trees were constructed with PhyML [14] and were edited with Mega v4.0 [15], [16]; node reliability was evaluated with 1,000 bootstraps. Tree labels were manually edited to make their size proportional to the sequence representation in the virus population. Trees were rooted at the most prevalent plasma V3-loop haplotype present before antiretroviral treatment initiation. Patterns of temporal and CXCR4-using clustering were investigated in subjects with 454 sequencing data available from the three compartments (plasma at T1 and PBMC at T1 and T2). The presence or absence of CXCR4-using clustering was only evaluated in subjects with at least two CXCR4-using viruses in any compartment. CXCR4-using clustering was defined as the presence of at least one cluster of at least two CXCR4-using haplotypes, supported by a bootstrap value or 70% or higher. Temporal clustering was defined as the grouping of all V3-loop haplotypes from one timepoint into a single cluster, supported by a bootstrap value or 70% or higher.

### Population differentiation

Population differentiation was assessed using the tree topology-based Slatkin-Maddison test [17] implemented in HYPHY [18]. The Slatkin-Maddison test compares tree topologies without taking into account haplotype frequency in the population. In previous controls, no compartmentalization was observed between duplicate measurements of 6 different PBMC samples by the Slatkin-Maddison test. However, an analysis of molecular variance (AMOVA) [19] provided statistically significant differences between duplicate samples, indicating that AMOVA can provide false positive results when deep sequencing data is used to estimate frequencies of closely related viral variants and, therefore, is not suitable for this analysis.

### Sequence Data Sets

V3-loop population and 454 sequences were deposited in GenBank (http://www.ncbi.nlm.nih.gov/genbank/index.html) and the Sequence Read Archive (http://www.ncbi.nlm.nih.gov/sra/), respectively, accession numbers: JF297475-JF297561 and SRP018530. Descriptions of biological source materials used in experimental assays are available in the Biosample database (http://www.ncbi.nlm.nih.gov/biosample) under consecutive accession numbers SAMN01914993 to SAMN01915082. The BioProject (http://www.ncbi.nlm.nih.gov/bioproject) code for this work is PRJNA188778.

## Results

### Subjects' characteristics

Thirty-five chronically HIV-1-infected adults were recruited for this study in Badalona, Spain, between June and October 2008. Subjects had to have stored samples available for tropism testing within 6 months before ART initiation (baseline, T1) and after at least 2 years of undetectable viremia (T2). (Figure 1) Five individuals were excluded from the analysis due to non-interpretable ESTA results (n = 1), treatment interruption during follow-up (n = 1), lack of amplification by any genotypic method (n = 1) and absence of sufficient sample material for testing (n = 2). The median age of the 30 individuals providing data was 44 years; they were mostly men and had acquired HIV through sexual practices (Table 1). The median time between T1 and T2 were 45 months. The median T1 viremia and CD4+ counts were 58,500 copies/mL and 224 cells/mm^3^, respectively; median nadir CD4+ counts were 215 cells/mm^3^. Median CD4+ counts increased to 560 cells/mm^3^ at T2 while HIV-1 RNA levels remained <50 copies/mL. Five individuals developed one HIV-1 RNA blip each during follow-up (HIV-1 RNA range: 125–275 copies/mL, incidence rate 1.7 blips/1,000 person-years). 454 sequencing produced a median (interquartile range, IQR) number of total, valid and unique V3-loop sequences of 5,366 (3,228 ; 6,553), 4,591 (2,751 ; 5,371) and 7 (4 ; 15) in plasma at T1; 3,983 (3,042 ; 5,256), 3,315 (2,238 ; 3,963) and 8 (6 ; 14) in PBMCs at T1; and 3,498 (2,444 ; 4,512), 2,562 (1,718 ; 3,509) and 8 (6 ; 15) in PBMCs at T2, respectively. All subjects were infected with subtype B HIV.

**Table 1:** Subjects' Characteristics.^a^

Age, median (IQR)	44 (39; 49)
Gender, n (%)	
Male	20 (67.7)
Female	10 (33.3)
Transmission route, n (%)	
Heterosexual	11 (36.7)
Homosexual	11 (36.7)
IVDU	2 (6.7)
IVDU + Homosexual	1 (3.3)
Transfusion of blood derivatives	1 (3.3)
Unknown	4 (13.3)
Pre-treatment HIV-1 RNA copies/mL, median (IQR)	58,500 (7,925; 107,500)
Pre-treatment CD4+ T cell count (cells/mm^3^)	
Absolute, median (IQR)	224 (120; 326)
Percentage, median (IQR)	15 (10; 19)
Nadir, median (IQR)	216 (86; 267)
CD4+ T cell count (cells/mm^3^) after >2 years of viremia suppression	
Absolute, median (IQR)	560 (416; 788)
Percentage, median (IQR)	29 (25; 37)
Pre-treatment CD8+ T cell count (cells/mm^3^)	
Absolute, median (IQR)	886 (657; 1,196)
Percentage, median (IQR)	41 (38; 51)
CD8+ T cell count (cells/mm^3^) >2 years of viremia suppression	
Absolute, median (IQR)	959 (665; 1,120)
Percentage, median (IQR)	62 (56; 71)
Antiretroviral treatment initiated, n (%)	
2 NRTIs + PIr	14 (46.7)
2 NRTIs + NNRTI	12 (40)
2 NRTIs + NNRTI + PIr	3 (10)
2 NRTIs	1 (3.3)
Time in months between events	
HIV diagnosis and T1, median (IQR)	2 (1;8)
T1 and T2, median (IQR)	45 (32;72)
ART initiation and T2, median (IQR)	33 (25; 51)

a IQR, 25^th^–75^th^ interquartile range; ART, Antiretroviral treatment; IVDU, Intravenous Drug User; NRTI, Nucleoside reverse transcriptase inhibitor; NNRTI, Non-nucleoside reverse transcriptase inhibitor; PIr, Ritonavir-boosted Protease Inhibitor; T1, first timepoint when tropism was measured (i.e. before ART initiation); T2, second timepoint when tropism was measured (i.e., after >2 years of HIV-1 RNA suppression).

### Accuracy of tropism assays relative to ESTA

The accuracy of tropism tests in plasma and PBMCs was only evaluated before ART initiation, when all assays could be simultaneously compared to the technical reference standard in the same clinical conditions. (Table 2)

**Table 2:** Accuracy of Tropism Assays Relative to the Enhanced-Sensitivity Trofile™ Assay.^a^

	Tests performed in pre-treatment Plasma RNA							Tests performed in pre-treatment Proviral DNA							
	Population V3-loop sequencing^b^			454 V3-loop sequencing				Population V3-loop sequencing^b^			454 V3-loop sequencing				MT-2^c^
G2P FPR^b^	20	10	5.75	15	10	5.75	3.5	20	10	5.75	15	10	5.75	3.5	
Sensitivity	60	40	40	72.7	72.7	63.6	45.5	63.6	36.4	36.4	72.7	72.7	54.5	45.5	45.5
	(26.2–87.4)	(12.1–73.8)	(12.1–73.8)	(39–94)	(39–94)	(30.8–89.1)	(16.7–76.6)	(30.8–89.1)	(10.9–69.2)	(10.9–69.2)	(39–94)	(39–94)	(23.4–83.3)	(16.7–76.6)	(16.7–76.6)
Specificity	100	100	94.4	84.2	94.7	100	100	100	100	100	73.7	73.7	84.2	89.5	100
	(74–100)	(74–100)	(74–100)	(60.4–96.6)	(74–99.9)	(75.1–100)	(75.1–100)	(75.1–100)	(75.1–100)	(75.1–100)	(48.8–90.9)	(48.8–90.9)	(60.4–96.6)	(66.9–98.7)	(75.1–100)
PPV^d^	100	100	100	72.7	88.9	100	100	100	100	100	61.5	61.5	66.7	71.4	100
	(42.1–100)	(28.4–100)	(28.4–100)	(30–94)	(51.8–99.7)	(47.3–100)	(35.9–100)	(47.3–100)	(28.4–100)	(28.4–100)	(31.6–86.1)	(31.6–86.1)	(29.9–92.5)	(29–96.3)	(35.9–100)
NPV^e^	81.8	75	75	84.2	85.7	82.6	76	82.6	73.1	73.1	82.4	82.4	76.2	73.9	76
	(59.7–94.8)	(53.3–90.2)	(53.3–90.2)	(60.4–96.6)	(63.7–97)	(61.2–95)	(54.9–90.6)	(61.2–95)	(52.2–88.4)	(52.2–88.4)	(56.6–96.2)	(56.6–96.2)	(52.8–91.8)	(51.6–89.8)	(54.9–90.6)
Accuracy^f^	85.7	78.6	78.6	80	86.7	86.7	80	86.7	76.7	76.7	73.3	73.3	73.3	73.3	80
	(67.3–96)	(59–91.7)	(59–91.7)	(61.4–92.3)	(69.3–96.2)	(69.3–96.2)	(61.4–92.3)	(69.3–96.2)	(57.7–90.1)	(57.7–90.1)	(54.1–87.7)	(54.1–87.7)	(54.1–87.7)	(54.1–87.7)	(61.4–92.3)

a Values are mean percentages (95% confidence interval of the mean), calculated assuming a binomial distribution of the data.

b *G2P FPR*, Geno2Pheno_[coreceptor]_ false positive rate used for population and 454 sequencing to assign CXCR4 use . The Geno2Pheno_[coreceptor]_ clonal model was always used.

c *MT-2*, Direct cocultivation of patient-derived peripheral blood mononuclear cells with MT-2 cells.

d *PPV*, Positive Predictive Value.

e *NPV*, Negative Predictive Value.

f “Accuracy” is defined as: (True positives + True negatives)/Total.

454 sequencing of plasma viruses provided the best agreement with ESTA, particularly at a Geno2Pheno_[coreceptor]_ False Positive Rate (FPR) of 10% (73% sensitivity, 95% specificity, 87% accuracy). Increasing the FPR to 15% did not improve the sensitivity but worsened the specificity; decreasing the FPR improved the specificity to 100%, but decreased the sensitivity to levels overlapping those of population sequencing. (Figure 1, Table 2) Interestingly, 454 sequencing was less accurate in proviral DNA than in plasma HIV-1 RNA, being similarly sensitive but less specific. Increases in specificity could be achieved by decreasing the FPR cut-off, but this also decreased the assay's sensitivity considerably. Moreover, the PBMC X4 load in subjects with detectable CXCR4-using HIV in PBMCs by 454 but R5 HIV-1 by ESTA (Table 3, subjects 13, 14, 19, 21 and 23) was usually high enough to suggest a different HIV population structure in plasma and PBMCs, rather than fluctuations around the sensitivity threshold of each technology amenable to fine-tuning.

**Table 3:** Longitudinal tropism testing results per subject.^a^,^b^,^c^

Subject ID	Before ART initiation (T1)								≥2 years of HIV-1 RNA suppression (T2)			
	HIV-1 RNA (cop/mL)	CD4+ count (c/mm^3^)	Tropism in Plasma RNA			Tropism in Proviral DNA			CD4+ count (c/mm^3^)	Tropism in Proviral DNA		
			ESTA	Pop Seq	454 (% X4)	Pop Seq	454 (% X4)	MT2		Pop Seq	454 (% X4)	MT2
1	300,000	191	DM	X4	100	X4	100	SI	326	X4	29	SI
2	60,000	88	DM	X4	100	X4	98.1	SI	272	X4	30.4	-
3	29,000	55	DM	X4	100	X4	90.1	SI	904	X4	79.4	SI
4	130,000	345	DM	X4	67.7	X4	93.9	SI	655	X4	100	SI
5	1,300,000	82	DM	-	12.2	-	15.7	SI	368	X4	16	-
6	40,000	231	DM	-	1.8	X4	68.9	-	950	X4	61.5	-
7	6,800	262	DM	-	2.8	-	-	-	528	X4	19.3	-
8	52,000	259	DM	X4	7.6	X4	15.4	-	872	X4	22.6	-
9	65,000	214	DM	X4	-	X4	1.8	-	471	X4	-	-
10	80,000	35	DM	NA	NA	-	-	-	488	-	-	-
11	89,000	379	DM	-	-	-	-	-	684	-	18.1	-
12	65,000	213	-	-	16.3	-	-	-	502	-	-	-
13	88,000	614	-	NA	-	-	99	-	1,384	NA	NA	-
14	2,300	265	-	-	-	-	36	-	665	-	3.3	-
15	1,220	418	-	-	-	-	-	-	760	-	-	-
16	1,290	281	-	-	-	-	-	-	298	-	-	-
17	57,000	539	-	-	-	-	-	-	1,232	-	-	-
18	860,000	52	-	-	-	-	-	-	608	-	-	-
19	180,000	131	-	-	-	-	11.4	-	1,056	-	-	-
20	226,195	182	-	-	-	-	-	-	490	-	-	-
21	1,600	64	-	-	-	-	7.3	-	283	-	1.2	-
22	1,000	331	-	-	-	-	-	-	1,001	NA	-	-
23	1,400	335	-	-	-	-	1.1	-	590	-	-	-
24	100,000	325	-	-	-	-	-	-	420	-	-	-
25	8,300	217	-	-	-	-	-	-	529	-	-	-
26	200,000	284	-	-	-	-	-	-	614	-	1.3	-
27	87,000	269	-	-	-	-	-	-	673	-	-	-
28	43,000	184	-	-	-	-	-	-	524	-	-	-
29	16,000	158	-	-	-	-	-	-	403	-	-	-
30	32,000	39	-	-	-	-	-	-	382	-	1.9	-

a *ESTA*, *Enhanced-Sensitivity Trofile™ Assay*; *Pop Seq*, population sequencing of the V3-loop; *454*, 454 sequencing of the V3-loop; *MT2*, direct co-cultivation of patient-derived peripheral blood mononuclear cells with MT-2 cells. HIV-1 RNA levels are in copies/mL; CD4+ cell counts are in cells/mm^3^.

b Population and 454 sequencing data shown here used the Geno2Pheno_[coreceptor]_ false positive rate cut-off providing highest accuracy when assigning HIV-1 tropism, i.e.: 20% and 10%, respectively. Based on internal error controls, only V3 forms present in ≥0.6% of viruses were considered for tropism prediction with 454 sequencing.

c Tests detecting CXCR4-using HIV are reported as “*dual-mixed, DM*” for ESTA, *“X4”* for population sequencing, “*percent of X4 viruses”* for 454, and “*syncytium-inducing, SI*” for MT-2 assays; for clarity, viruses only using CCR5 are shown as dashes; NA, tropism test result not available due to lack of amplification.

Population sequencing was invariably less sensitive than 454 sequencing although it retained high specificity. No differences in accuracy were observed using either 10% or 5.75% FPR cut-offs. However, increasing the FPR threshold to 20% improved the sensitivity of the assay without compromising its specificity. Of note, population sequencing achieved similar accuracy in proviral DNA than in plasma HIV-1 RNA. All subjects with CXCR4-using viruses by population sequencing except one had CXCR4-using virus levels above 15% by 454 sequencing in proviral DNA, further supporting the idea that clinically relevant CXCR4-using cut-off levels might be different for plasma and PBMCs.

The MT-2 assays had low sensitivity but high specificity. As observed elsewhere [20], internal controls of direct cocultivation of patient and healthy donor PBMCs indicated that we were only capable of obtaining productive HIV infection from about 50% of subjects overall (not shown).

The prevalence of subjects with a CXCR4-using virus varied among tropism tests and settings (Figure 1). ESTA detected CXCR4-using viruses in 36.7% of subjects. The prevalence of CXCR4-using HIV was significantly underestimated by plasma 454 sequencing using a FPR of 3.5%, population sequencing of plasma and PBMC V3 forms using FPRs of 10% or 5.75%, and by the MT-2 assay (p<0.05 for all comparisons with ESTA, two-sided exact binomial test). No statistically significant differences in CXCR4-using prevalence were observed between plasma and PBMCs.

### Viral evolution during viremia suppression and population differentiation

**Figure 2:**
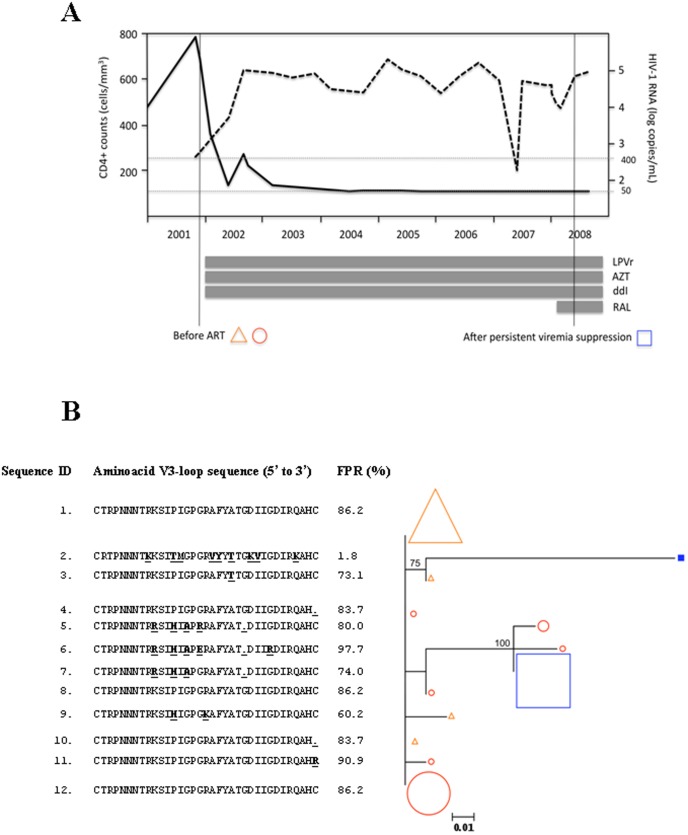
Selection of a CXCR4-using variant above the 454 sequencing error threshold during persistent viremia suppression in Subject 26. ***Panel A, antiretroviral treatment history, virological and immunological evolution*** **.** Continuous line, HIV-1 RNA levels; dashed line, CD4+ counts; horizontal bars, time period during which a given antiretroviral drug was prescribed. Vertical lines indicate the timepoints when 454 sequencing was performed. LPVr, lopinavir/ritonavir; AZT, zidovudine; ddI, didanosine; RAL, raltegravir. ***Panel B, maximum likelihood nucleotide-based phylogenetic tree*** including V3-loop haplotypes present at a frequency ≥0.6% in the virus population in plasma (triangles), PBMCs before therapy initiation (circles) and PBMCs after persistent viremia suppression (squares). The tree is rooted at the most frequent plasma sequence before antiretroviral treatment initiation. Filled symbols show predicted CXCR4-using viruses; open symbols show predicted CCR5-using viruses. Symbol size increases proportionally to the V3-loop haplotype frequency in the virus population in 10% intervals. Node reliability was tested using 1000 bootstraps; bootstrap values ≥50% are shown. The V3-loop aminoacid sequence translation is shown next to each taxon. Aminoacid changes relative to the predominant sequence in plasma are highlighted in bold and underlined. Gaps correspond to aminoacid indeterminations. A Geno2Pheno _[coreceptor]_ false positive rate (FPR) equal or lower than 10% was used to define CXCR4 use. The actual false positive rate of each sequence is shown. *Sequence #2 was identical to one detected in 0.04% of PBMC-associated viruses, below the error threshold, before treatment initiation.

Overall, there was good individual agreement in the longitudinal detection of CXCR4-using HIV (Table 3), but slightly more CXCR4-using viruses were detected in PBMCs after at least two years of viremia suppression (Table 3, Figure 1). To evaluate if viruses evolved in PBMCs during suppressive ART, we used V3 forms generated by 454 sequencing present in ≥0.6% in the virus population. Based on the previous accuracy assessments, a FPR ≤10% was chosen to define CXCR4 use. Twenty-eight subjects had 454 sequencing data available from plasma at T1, PBMCs at T1 and PBMCs at T2 (Figure 2, File S1).

**Figure 3:**
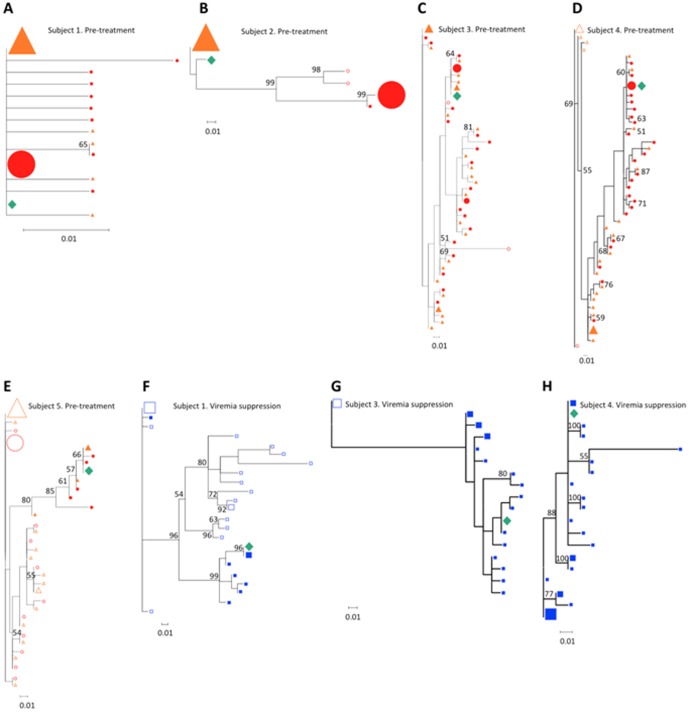
V3-loop haplotypes detected by quantitative deep sequencing are also found in CXCR4-using viruses growing in MT2 assays. Maximum likelihood phylogenetic trees showing that the V3-loop sequence of syncytium-inducing viruses grown in MT-2 assays (diamond) is identical to one of the predominant V3-loop haplotypes detected with quantitative deep sequencing in proviral DNA and/or plasma RNA before antiretroviral therapy initiation (Trees A to E) or after at least 2 years of persistent viremia suppression (trees F to H). Trees include V3-loop haplotypes present at a frequency ≥0.6% in the virus population in plasma (triangles), PBMCs before therapy initiation (circles) and PBMCs after persistent viremia suppression (squares); trees are rooted at the predominant plasma (trees A to E) or PBMC (trees G to H) V3-loop haplotype. Filled symbols represent CXCR4-using viruses; open symbols show R5 viruses. Symbol size increases proportionally to the V3-loop haplotype frequency in the virus population in 10% intervals. Node reliability was tested using 1,000 bootstraps; bootstrap values ≥50% are shown. CXCR4 use was defined by a Geno2Pheno _[coreceptor]_ false positive rate ≤10%.

We only observed evolution of CCR5 viruses in one individual (Subject 7), who showed bootstrap-supported temporal clustering of new CCR5 viruses at T2. Apparently, another subject seemed to have developed CXCR4-using HIV-1 *de novo* during viremia suppression (subject 26). The emerging CXCR4-using V3-loop form (sequence ID 2; Figure 2B), however, was present before therapy in 0.04% of PBMC-associated viruses, suggesting that detection of such CXCR4-using virus was more likely due to fluctuations around the sensitivity threshold of 454 sequencing than to a true emergence of a CXCR4-using virus *de novo*. Subject 30 (Table 3) also had low-frequency CXCR4-using HIV detected only at T2. Phylogenetic analysis (File S1) confirmed that such variant clustered with pre-treatment viruses. This indicates that this was a pre-existing which was detected at T2 due to fluctuations around the sensitivity threshold of 454 sequencing, rather than to true virus evolution. Of the remaining individuals, 9 (32.1%) showed bootstrap-supported CXCR4-using HIV-1 clustering across timepoints and compartments, 3 (10.7%) had non-significant CXCR4-using virus clustering and 15 (53.6%) had only R5 viruses detected in all timepoints and compartments. Interestingly, the V3-loop sequence of syncytium-inducing viruses grown in all positive MT-2 assays was identical to one of the predominant V3-loop haplotypes simultaneously detected by 454 sequencing in proviral DNA and/or plasma RNA (Figure 3), suggesting that 454 sequencing can detect V3 forms that are also present in functional CXCR4-using viruses.

There were no differences in nucleotide variability (π) among compartments in any subject. (Table 4) The Slatkin-Maddison test indicated the presence of sequence compartmentalization between plasma and PBMCs at T1 in 2/28 (7%) individuals, and between PBMCs at T1 and T2 in 6/28 (21%) individuals.

**Table 4:** Population structure analysis of plasma and PBMC V3 forms detected by 454 sequencing.^a^

Subject ID	Intracompartment Variability (Π)			Slatkin-Madison Test	
	Plasma T1	PBMC T1	PBMC T2	Plasma T1 vs PBMC T1	PBMC T1 vs PBMC T2
1	0.0156	0.0097	0.1469	2	2
2	NC	0.1190	0.1052	1	4
3	0.0351	0.0476	0.0457	18	7**
4	0.0645	0.0591	0.0276	13	13
5	0.0476	0.0606	0.0622	14	16
6	0.0271	0.0277	0.0307	4	4
7	0.0362	0.0293	0.0923	6	3*
8	0.0329	0.0286	0.0349	20	16
9	0.0130	0.0171	0.0779	3	1**
11	0.0116	0.0096	0.0274	4	3
12	0.1184	0.0170	0.0000	4*	2
14	0.0176	0.1509	0.0728	2**	6**
15	0.0156	0.0187	0.0163	4	4
16	0.1598	0.0169	0.0157	3	4
17	0.0167	0.0244	0.0230	3	8
18	0.0214	0.0208	0.0162	5	3
19	NC	0.0317	0.0605	1	2
20	0.0379	0.0286	0.0360	9	8
21	0.0264	0.0560	0.0472	4	10
22	0.0337	0.0342	0.0483	5	5
23	0.0425	0.0539	0.0424	12	2**
24	0.0130	0.0177	0.0163	3	4
25	0.1163	0.0225	0.0271	7	2**
26	0.0108	0.0306	0.1447	4	2
27	0.0129	0.0262	0.0262	2	6
28	0.0502	0.0552	0.0184	10	4
29	0.0222	0.0145	0.0182	4	4
30	0.0347	0.0321	0.0346	8	11

a The intracompartment variability (*Π*) of each sample is measured with the best evolutionary model found with *Findmodel* (www.hiv.lanl.gov); it corresponds to the average number of nucleotide differences per site between sequences. Migration events with *p-value*, and *F_ST_* with *p-value* are indicated for Slatkin-Madison population structure tests. *NA* indicates comparisons where the tests were not applicable. *NC* indicates that variability cannot be calculated because there is only one haplotype.

* p-value between 0.05 and 0.01;

** p-value<0.01 and 10^−6^.

Statistically significant p-values are colored; the color intensity is proportional to the p-value. Note that a complete dataset was not available for subjects 10 and 13, which were, therefore, not included in this analysis.

## Discussion

Only assays assessing tropism of plasma HIV-1 have been validated in clinical trials. Given that tropism cannot be routinely assessed in plasma of subjects with HIV-1 RNA levels <500–1,000 copies/mL, the optimal tropism testing strategy for clinical decision-making in these individuals remains unclear. To better understand the advantages and limitations of different tropism testing strategies in aviremic subjects, we first evaluated the diagnostic performance of various state-of-the-art tropism tests relative to the ESTA. The latter was chosen as the technical reference standard for this study because it is widely recognized as a sensitive and robust assay, it is CLIA-validated, and has been used in most previous clinical trials of CCR5 antagonists [3], [4], [5], [6], [21], including those leading to the approval of maraviroc [3], [4], [5].

In our hands, the tropism assay providing closest diagnostic accuracy to ESTA was 454 sequencing of plasma RNA. The highest accuracy of this assay was obtained after applying strict PCR and 454 sequencing error controls, considering only V3 forms present in at least 0.6% of the virus population and using a Geno2Pheno _[coreceptor]_ FPR cut-off of 10% to define CXCR4-using viruses. Such settings provided 73% sensitivity, 95% specificity and 87% accuracy. Using a 2% level of minor CXCR4-using variants and a 3.5% Geno2Pheno _[coreceptor]_ FPR to define non-R5 use, other authors found that 454 sequencing of plasma HIV-1 was able to accurately predict virological response to maraviroc-including regimens in retrospective reanalyses of maraviroc trials [4], [5], [6], [7]. However, applying such settings to our own dataset (including the assessment of tropism by Geno2Pheno_[454]_ starting from raw sequence data) resulted in decreased sensitivity and accuracy of the assay (50% sensitivity, 95% specificity and 79% accuracy). Consequently, the settings chosen for 454 sequencing in our study might potentially provide a more accurate assessment of phenotypic tropism; proper validation of our 454 settings in larger datasets including clinical endpoints is however warranted before they can be routinely used to predict virological outcomes to CCR5 antagonists.

454 sequencing was remarkably less accurate in proviral DNA than in plasma, mainly due to reduced specificity. In fact, population sequencing in proviral DNA was slightly less sensitive but more specific and, overall, more accurate than 454 sequencing in PBMCs. The reasons for such discrepancy are not fully understood. On one hand, tropism prediction engines have been trained and validated using data from plasma viruses, and might require different settings in other compartments. Peripheral blood mononuclear cells contain a historical repository of current and past HIV, some of which might be defective or unable to replicate. Also, identical V3 forms are often present at different frequency in plasma and PBMCs; whether this has an impact on treatment outcomes remains unclear. Even if identical V3 forms can be identified simultaneously in plasma RNA, proviral DNA and in syncytium-inducing viruses growing in MT-2 cells, as observed in this study, such V3 loops may be present in viruses with different genetic backgrounds and, thus, have different phenotypic tropism. Mutations in gp120 outside V3 loop and in gp41 [22], as well as differences in env glycosylation patterns [23] can modulate the viral tropism in a minority of subjects. This is a limitation of most genotypic techniques based on V3 loop sequencing. However, although mutations in gp41 are correlated with coreceptor tropism, they do not improve tropism prediction methods substantially [24].

The accuracy of population sequencing of plasma or PBMC-associated HIV in our study was similar to that of other studies that also used the ESTA as the reference standard [25], [26]. Interestingly, although population sequencing was less sensitive than plasma 454, it was more specific and, overall, just slightly less accurate, particularly when a 20% FPR was used. This suggests that population sequencing might be an acceptable alternative to 454 sequencing in settings without access to next-generation sequencing provided that high FPR cutoffs are used.

One particular interest of our study was to evaluate to which extent tropism tests in plasma and PBMCs provide equivalent biological results. The observation of sequence compartmentalization in some subjects suggests that, although plasma and PBMCs often provide similar tropism reports [27], their biological or clinical meaning might not be necessarily equivalent. Indeed, in a retrospective reanalysis of pretreatment samples of the MOTIVATE and A4001029 studies, HIV DNA-based methods were generally good predictors of virological response to maraviroc regimens, but virologic response was better predicted by plasma compared to PBMC 454 sequencing [28]. Contrasting with our dataset, the PBMC compartment harbored more variable V3 forms than plasma. Concordance between plasma and PBMC tropism by 454 sequencing ranged from 74% amongst samples with CD4+ counts <50 cells/mm^3^ to 100% concordance at CD4+ counts >350 cells/mm^3^, suggesting that, in addition to the sequence compartmentalization observed in our study, a CD4+ count-dependant bias in DNA input might also affect PBMC results. Moreover, the existence of V3 compartmentalization between PBMCs at T1 and T2 in the absence of overt viral evolution during prolonged viremia suppression suggests the presence of drifts in PBMC composition that may further affect DNA sampling for tropism testing.

We also sought to explore if HIV-1 tropism shifts were frequently observed during persistent viremia suppression, which would be informative of the clinical feasibility of using stored plasma samples collected before ART initiation for tropism testing. The absence of CXCR4 virus evolution during prolonged periods of viremia suppression suggests that, when available, testing of stored plasma samples is generally safe and informative, provided that HIV-1 RNA levels remain continuously suppressed. Our findings are in agreement with previous publications using population sequencing of proviral DNA [29], [30]. Importantly, subjects in this study did not receive CCR5 antagonists; it remains largely unexplored if HIV-1 tropism may still evolve under the selective pressure of CCR5 antagonists if continuous viremia suppression is achieved.

This study represents a comprehensive comparison of the main state-of-the-art tropism tests with potential application to HIV clinical management and fulfills the Standards for Reporting of Diagnostic Accuracy (STARD) [31]. The study design allowed investigating population differentiation and longitudinal CXCR4-using evolution in different compartments, which is informative of the optimal timing and source for tropism testing in subjects with undetectable HIV-1 RNA levels. The main weaknesses of the study are its small sample size, its retrospective nature, the lack of association of tropism data with outcomes to CCR5 antagonist therapy, and the use of cryopreserved PBMCs for MT-2 assays. This last factor, and the fact that subjects had had HIV RNA <50 copies/mL during more than 2 years of ART at T2, likely reduced our ability to recover infectious viruses, leading to a decreased performance of MT2 assays relative to previous publications [32]. We only used Geno2Pheno_[coreceptor]_ to interpret genotypic data because previous comparisons demonstrated equivalence with other interpretation systems [33], [34] and it is extensively used in our setting. Differences in FPR settings observed in this study were sometimes due to a small number of patients, which is reflected in the wide confidence intervals of our diagnostic accuracy estimations.

## Conclusions

Although plasma and PBMCs may often provide similar tropism reports, [27] tropism testing in PBMCs may not necessarily produce equivalent biological results to plasma, because the structure of viral populations and the diagnostic performance of tropism assays vary between compartments. Thereby, proviral DNA tropism testing should be specifically validated in clinical trials before it can be applied to routine clinical decision-making. Clinically relevant cut-offs and settings should be identified for clonal and population genotypic tropism testing in proviral DNA. The absence of tropism shifts during viremia suppression suggests that, when available, testing of stored plasma samples is generally safe and informative, provided that HIV suppression is maintained. Next-generation sequencing technologies have the potential to be a cost-effective alternative to assess viral tropism and provide essential information to increase the efficacy of HIV therapeutics and advance our understanding of HIV pathogenesis.

## Supporting Information

File S1 **Phylogenetic relatedness of V3 forms before treatment initiation and after 2 years in each subject.** Maximum-likelihood phylogenetic trees including V3-loop haplotypes present at a frequency ≥0.6% in the virus population in plasma (triangles), PBMCs before therapy initiation (circles) and PBMCs after persistent viremia suppression (squares). One tree is shown per each subject. Trees are rooted at the most frequent plasma sequence before antiretroviral treatment initiation. Filled symbols show predicted CXCR4-using viruses; open symbols show predicted CCR5-using viruses. Symbol size increases proportionally to the V3-loop haplotype frequency in the virus population in 10% intervals. Node reliability was tested using 1000 bootstraps; bootstrap values ≥50% are shown. A Geno2Pheno _[coreceptor]_ false positive rate (FPR) equal or lower than 10% was used to define CXCR4 use.
(PDF)
