# Supplementary material for: HIV-1 Tropism Testing in Subjects Achieving Undetectable HIV-1 RNA: Diagnostic Accuracy, Viral Evolution and Compartmentalization
Source: PLoS One. 2013 Aug 1;8(8):e67085. doi: 10.1371/journal.pone.0067085 (PMC3731261; doi:10.1371/journal.pone.0067085)
Supplement: File S1 — Phylogenetic relatedness of V3 forms before treatment initiation and after 2 years in each subject. Maximum-likelihood phylogenetic trees including V3-loop haplotypes present at a frequency ≥0.6% in the virus population in plasma (triangles), PBMCs before therapy initiation (circles) and PBMCs after persistent viremia suppression (squares). One tree is shown per each subject. Trees are rooted at the most frequent plasma sequence before antiretroviral treatment initiation. Filled symbols show predicted CXCR4-using viruses; open symbols show predicted CCR5-using viruses. Symbol size increases proportionally to the V3-loop haplotype frequency in the virus population in 10% intervals. Node reliability was tested using 1000 bootstraps; bootstrap values ≥50% are shown. A Geno2Pheno [coreceptor] false positive rate (FPR) equal or lower than 10% was used to define CXCR4 use. (PDF) [file pone.0067085.s001.pdf]

Supplementary File S1: Phylogenetic analyses of all subjects

HIV-1 Tropism Testing in Subjects Achieving Undetectable HIV-1 RNA: Diagnostic Accuracy, Viral Evolution and Compartmentalization

Christian Pou[1]*, Francisco M. Codoñer[1], Alexander Thielen[3], Rocío Bellido[1], Susana Pérez-Álvarez[1], Cecilia Cabrera[1], Judith Dalmau[1], Marta Curriu[1], Yolanda Lie[5], Marc Noguera[1], Jordi Puig[2], Javier Martinez-Picado[1,4], Julià Blanco[1], Eoin Coakley[5], Martin Däumer[6], Bonaventura Clotet[1,2], Roger Paredes[1,2]*.

[1]Institut de Recerca de la SIDA irsiCaixa - HIVACAT & [2]HIV Unit-Fundació Lluita contra la SIDA, Hospital Universitari Germans Trias i Pujol, Universitat Autònoma de Barcelona, Catalonia, Spain; [3]Max-Planck-Institut für Informatik, Saarbücken, Germany; [4]Institució Catalana de Recerca i Estudis Avançats (ICREA), Barcelona, Spain; [5]Monogram Biosciences Inc., South San Francisco, California, USA, [6]Institut für Immunologie und Genetik, Kaiserlautern, Germany.

Maximum-likelihood phylogenetic trees were constructed for each subject using PhyML and the best nucleotide evolution model identified by Modeltest. Trees included V3-loop haplotypes present at a frequency ≥0.6% in the virus population in plasma (triangles), PBMCs before therapy initiation (circles) and PBMCs after persistent viremia suppression (squares). One tree is shown per each subject. Trees are rooted at the most frequent plasma sequence before antiretroviral treatment initiation. Filled symbols show predicted CXCR4-using viruses; open symbols show predicted CCR5-using viruses. Symbol size increases proportionally to V3-loop haplotype frequency in the virus population in 10% intervals. Node reliability was tested using 1000 bootstraps; bootstrap values ≥ 50% are shown. A Geno2Pheno $_{[coreceptor]}$ false positive rate (FPR) equal or lower than 10% was used to define CXCR4 use.

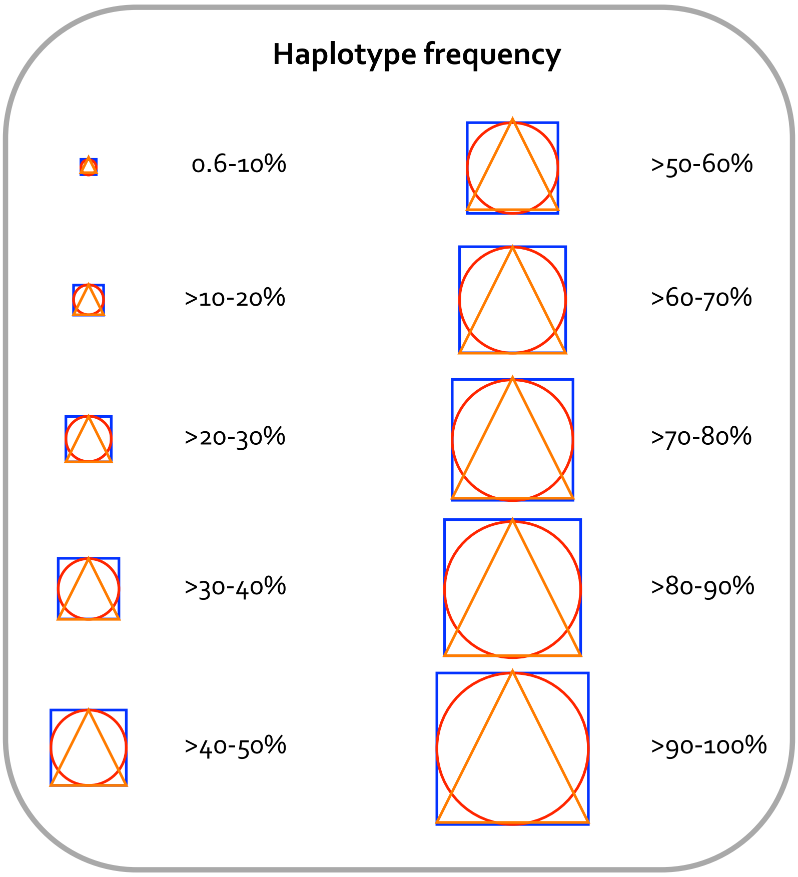

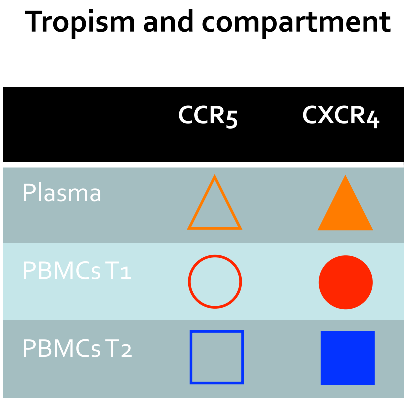

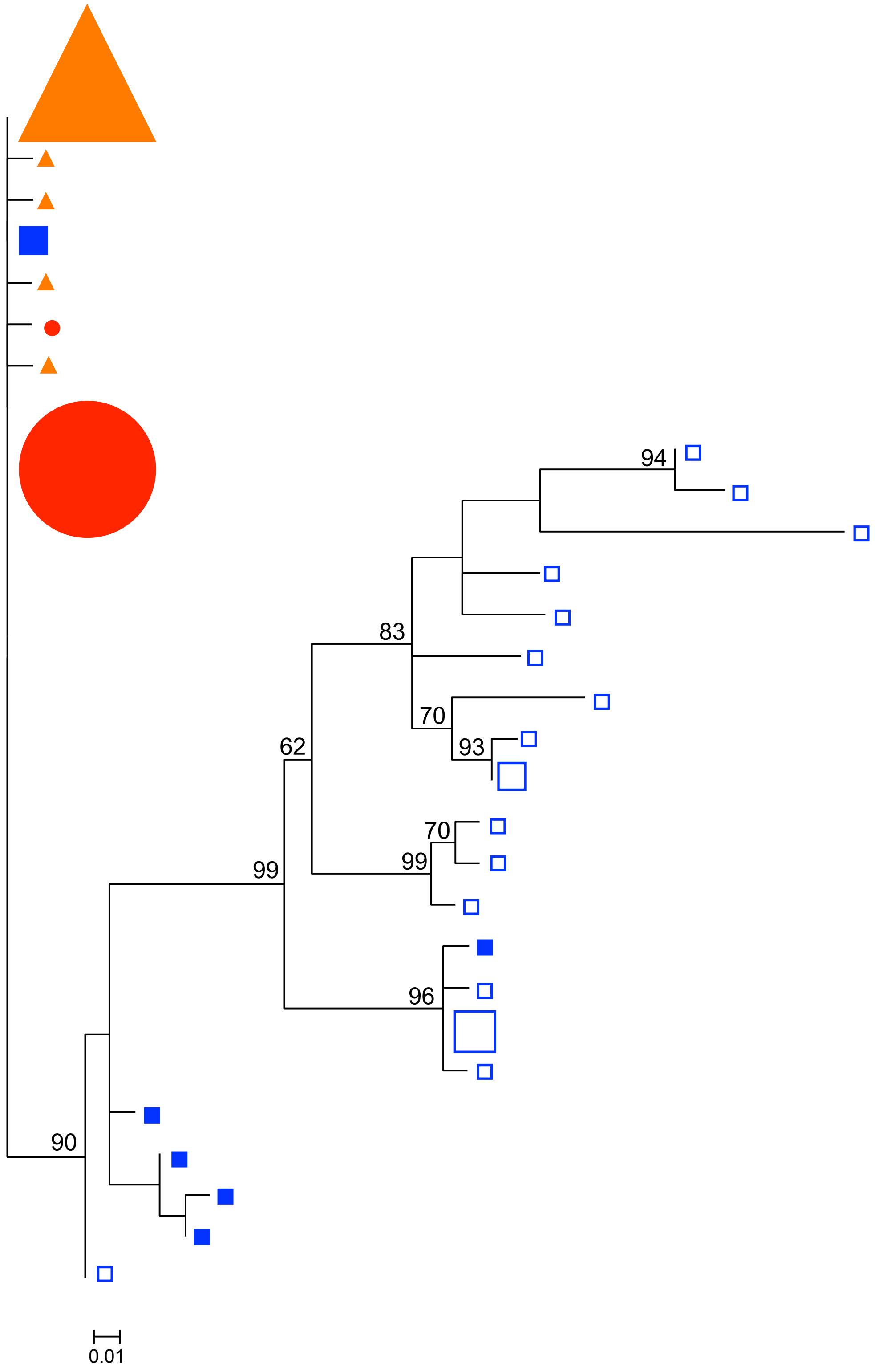

Subject 1

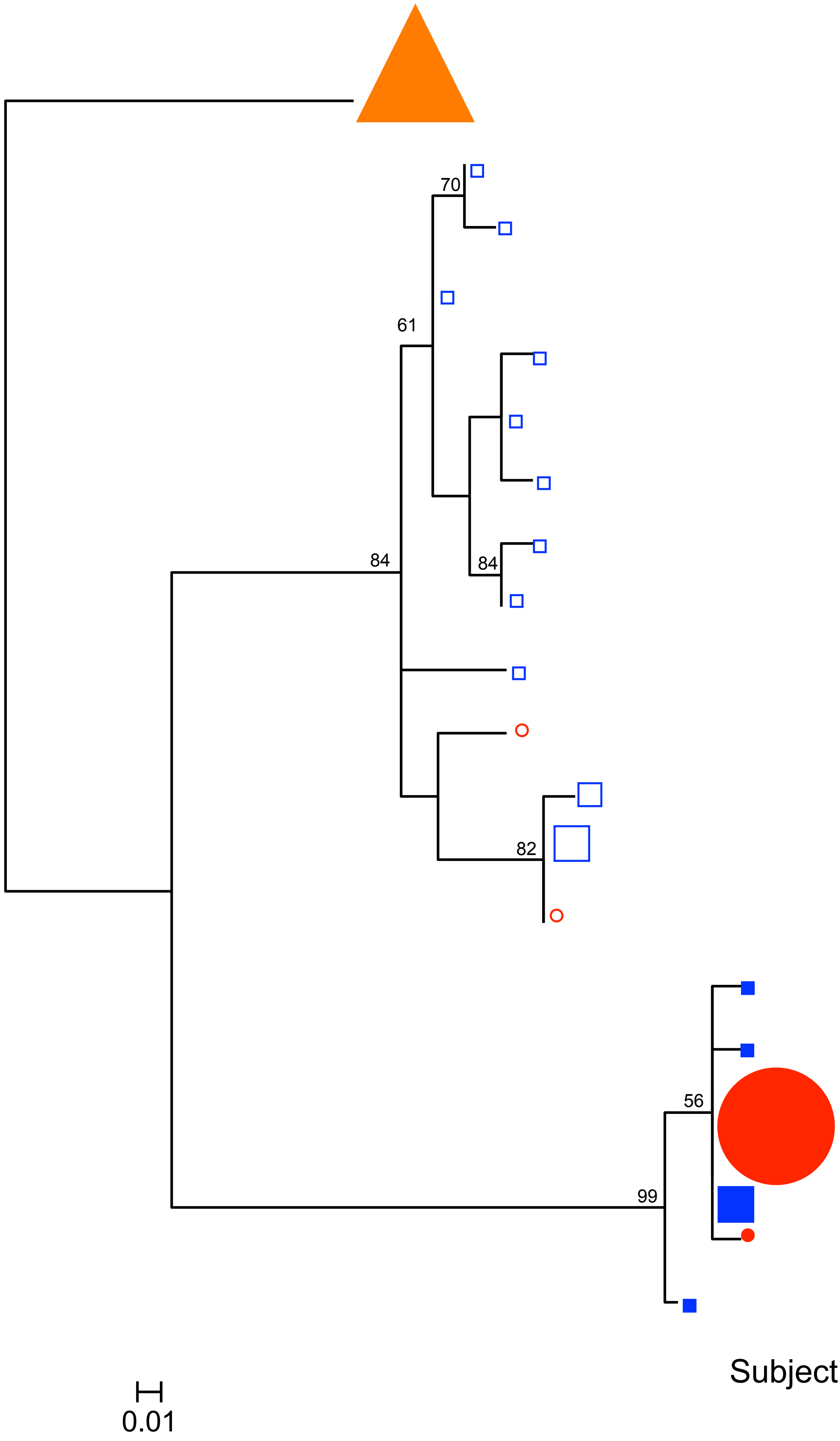

Subject 2

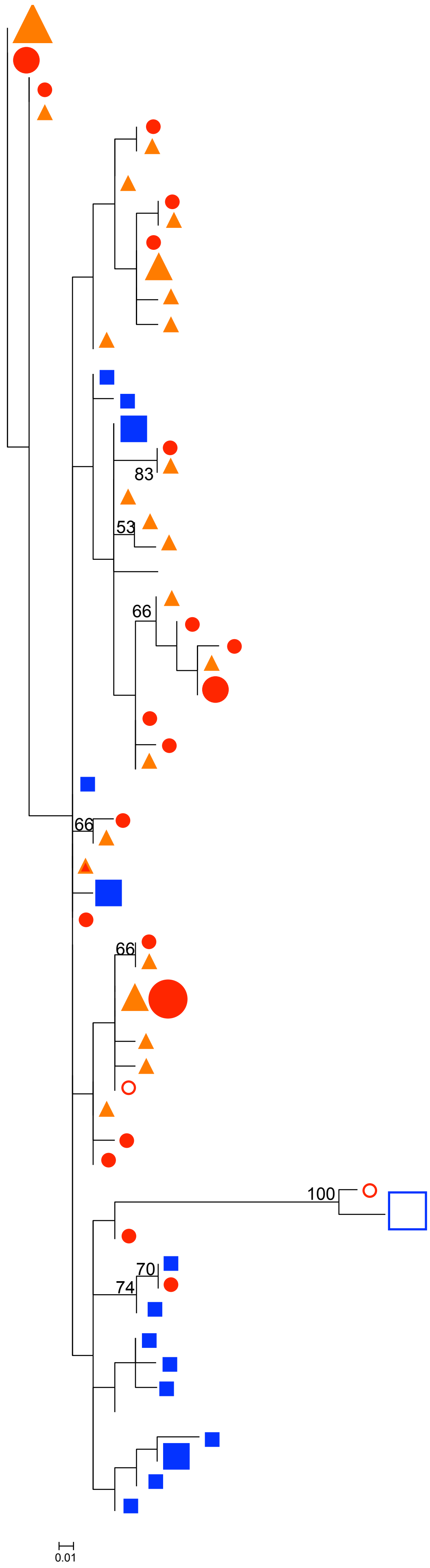

Subject 3

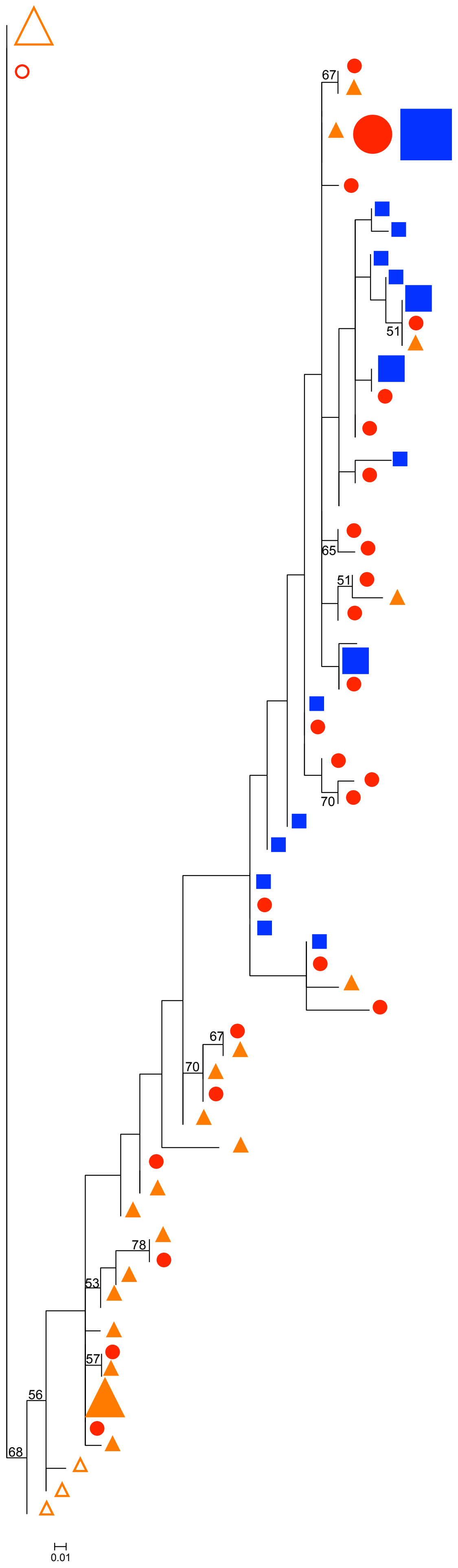

Subject 4

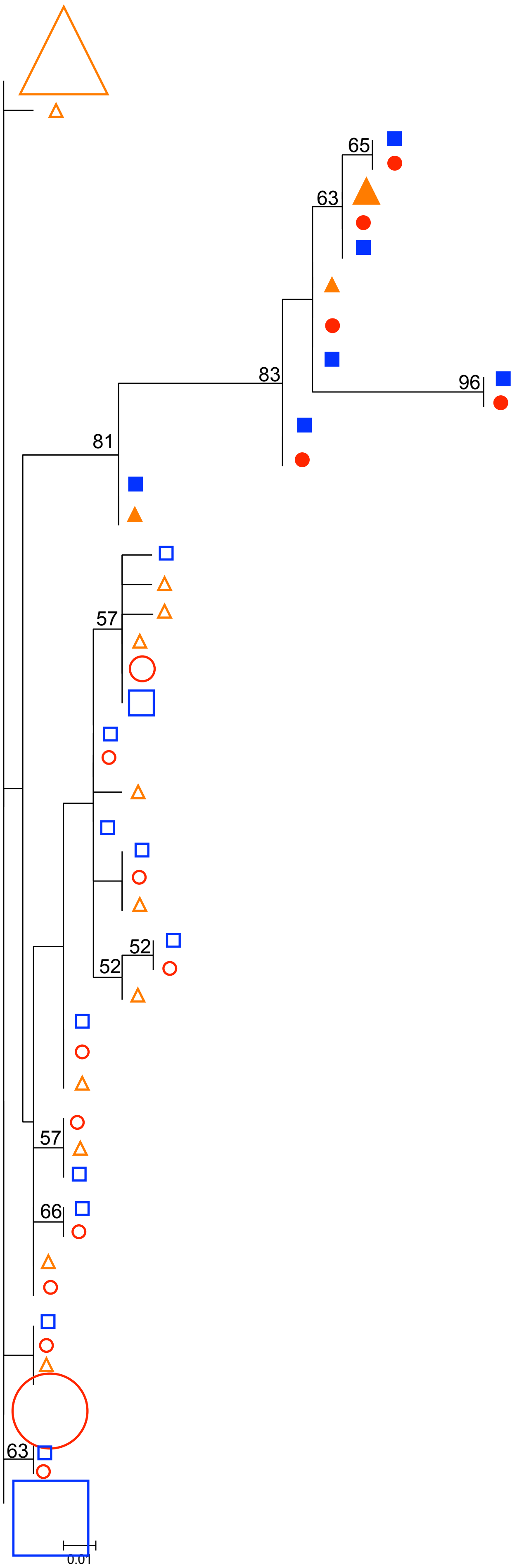

Subject 5

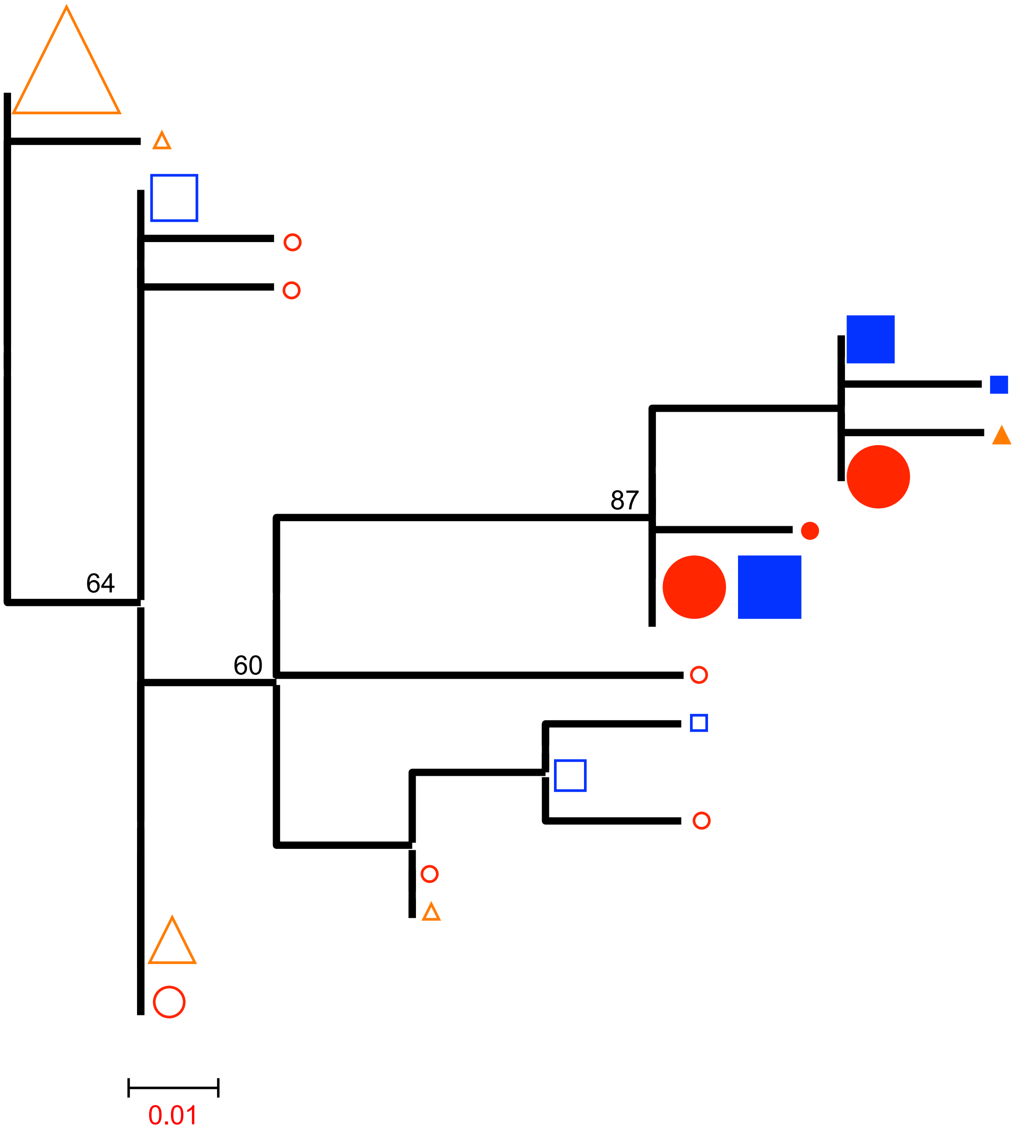

Subject 6

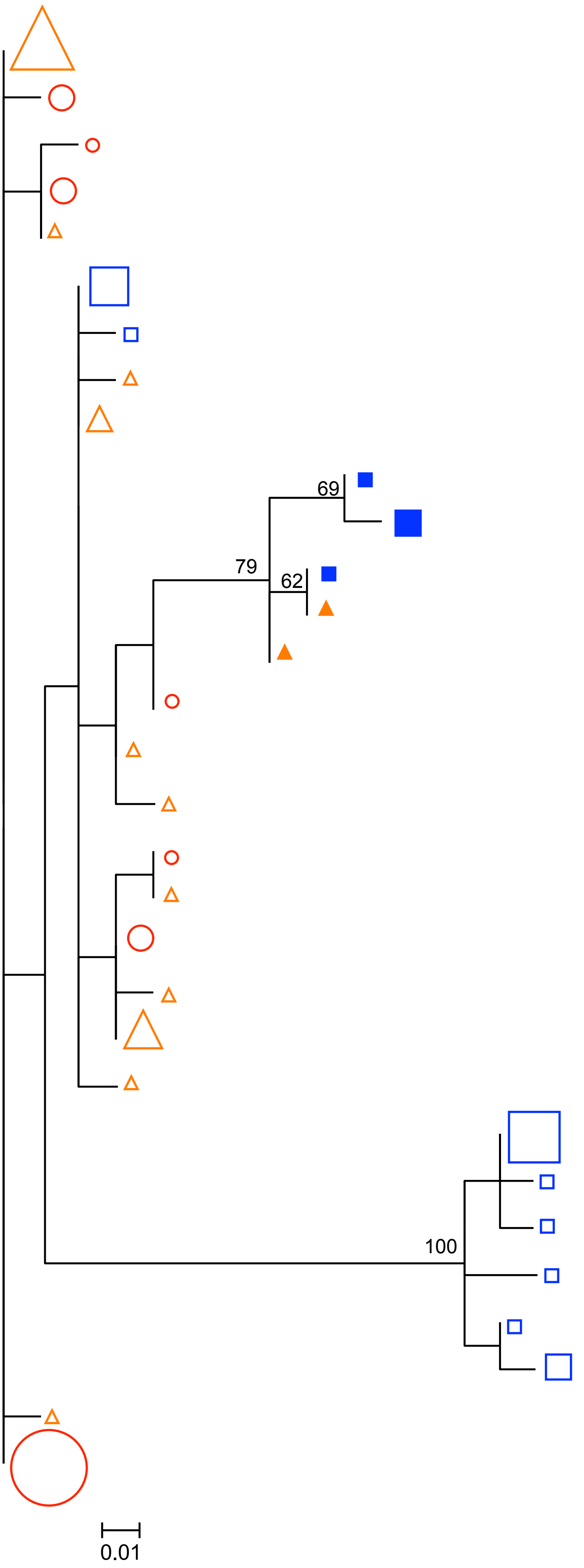

Subject 7

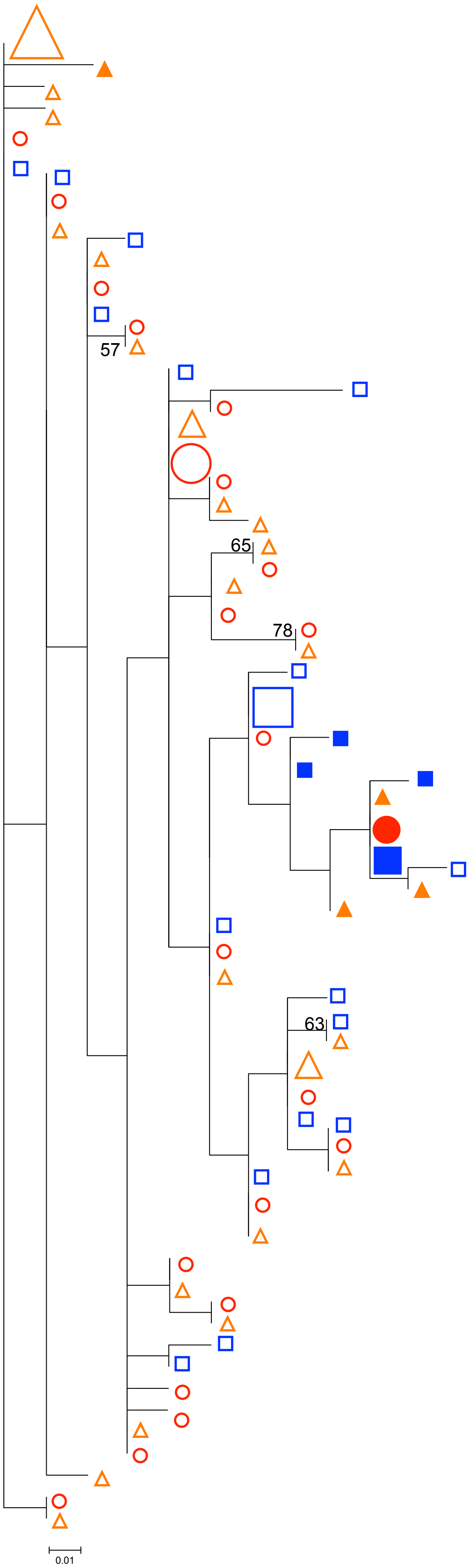

Subject 8

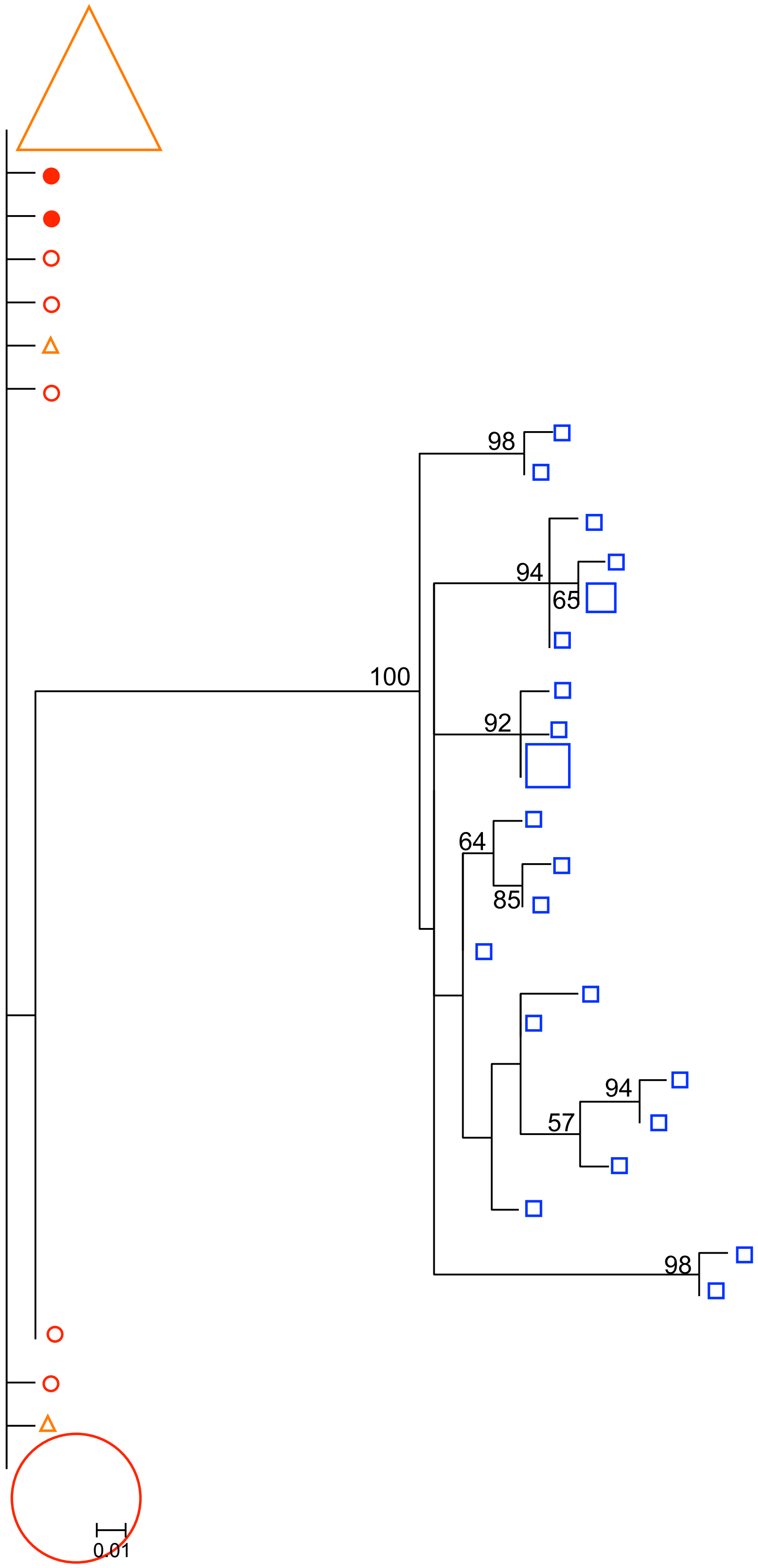

Subject 9

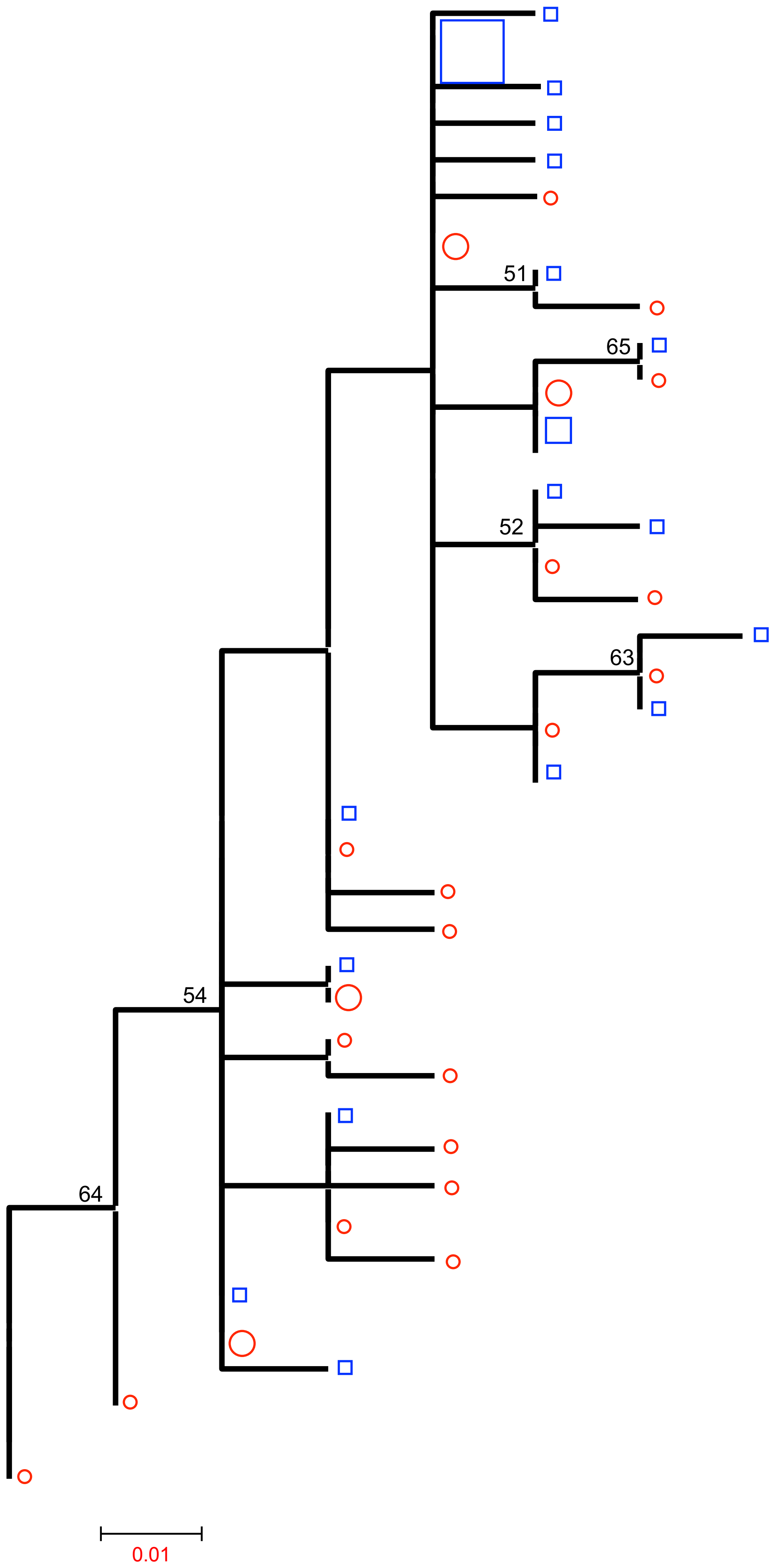

Subject 10

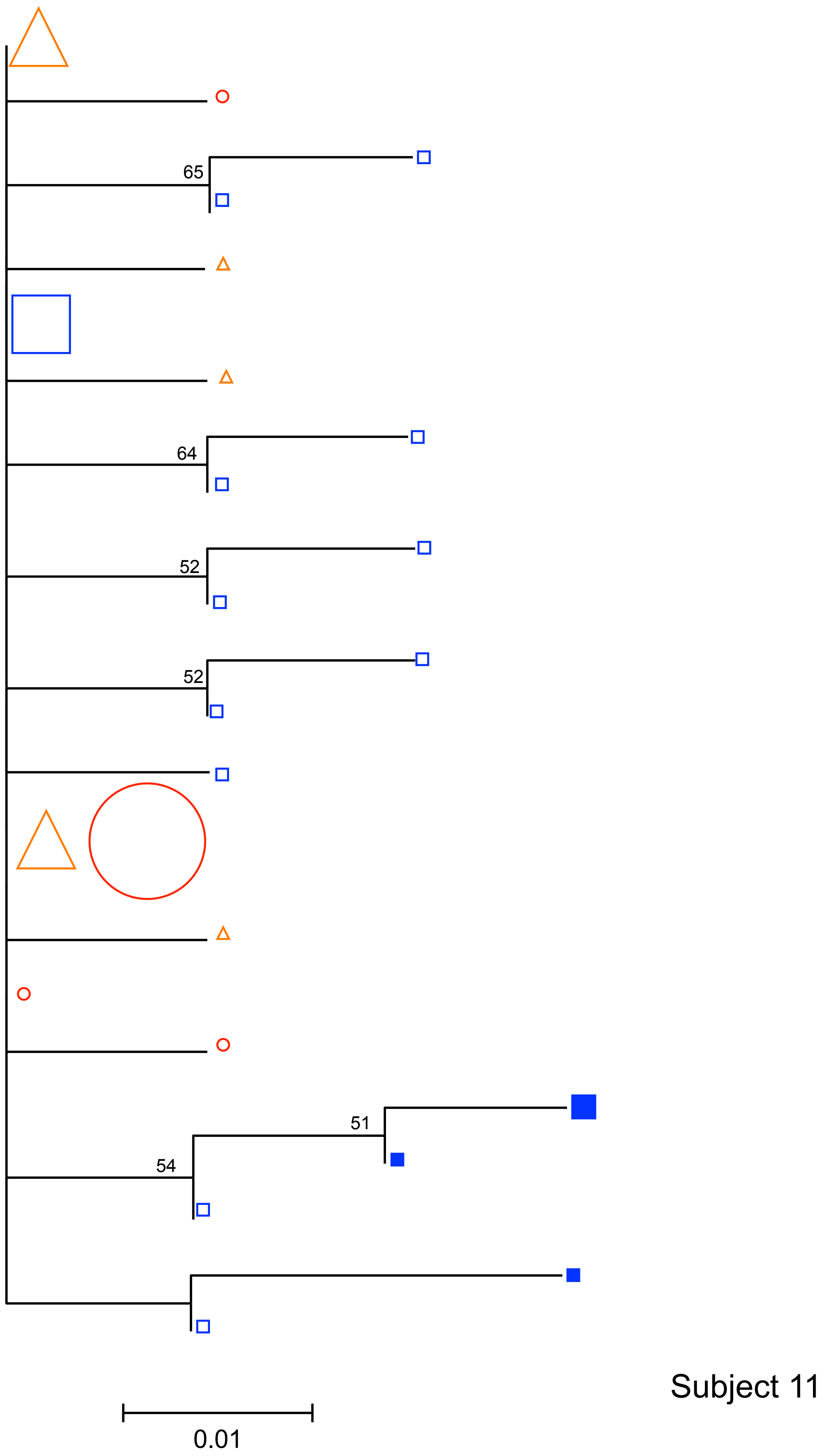

65
64
52
52
51
54
0.01
Subject 11

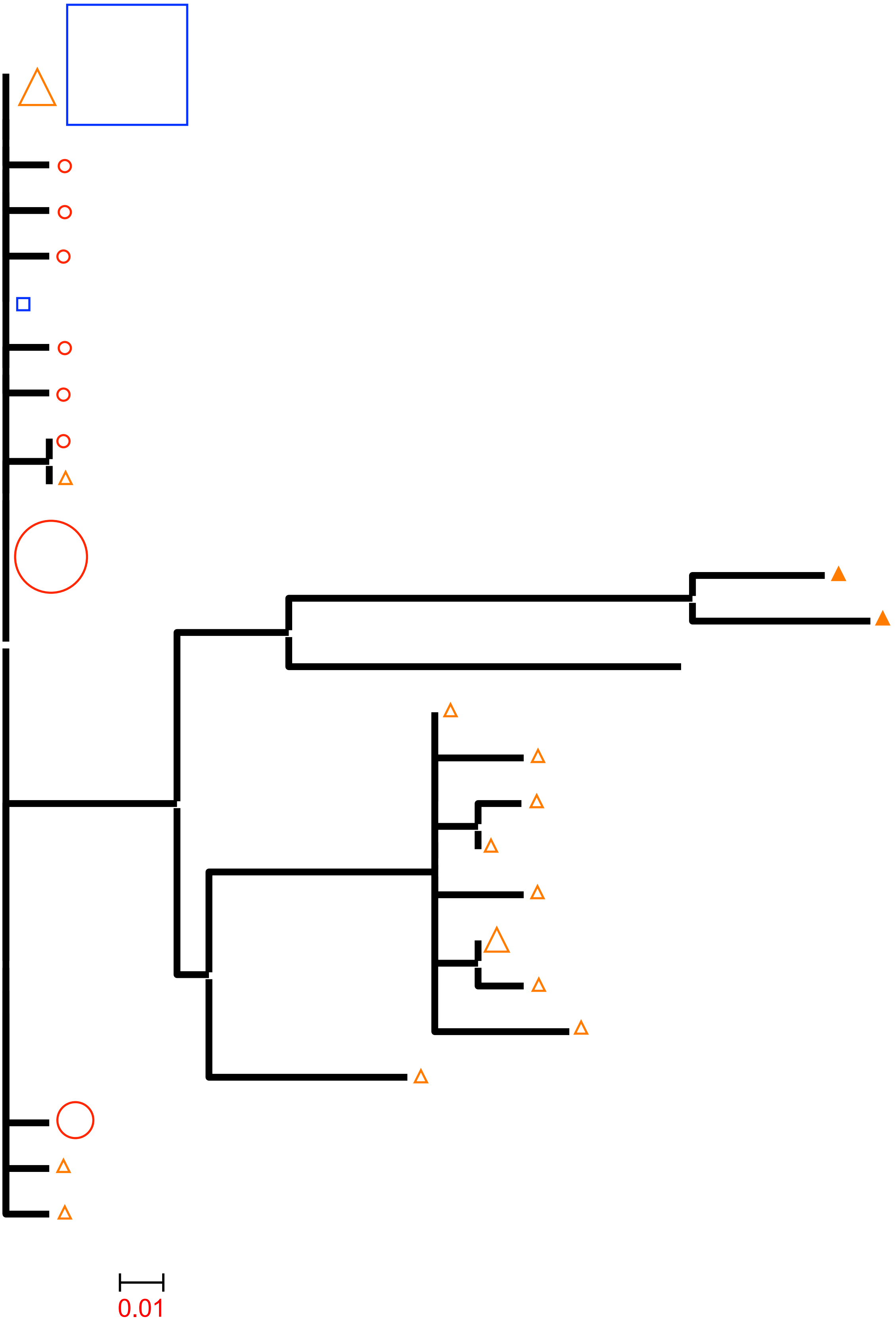

Subject 12

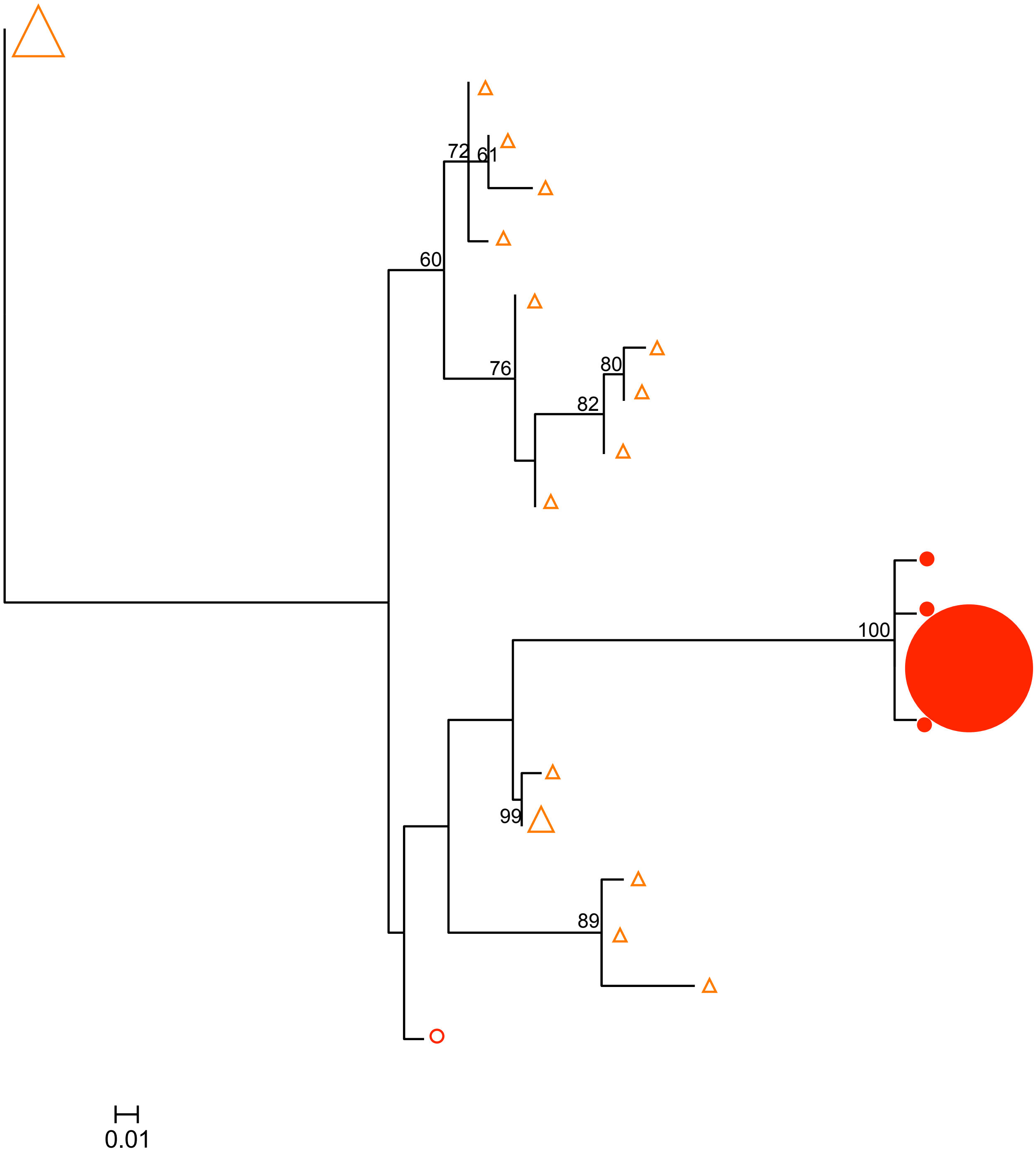

Subject 13

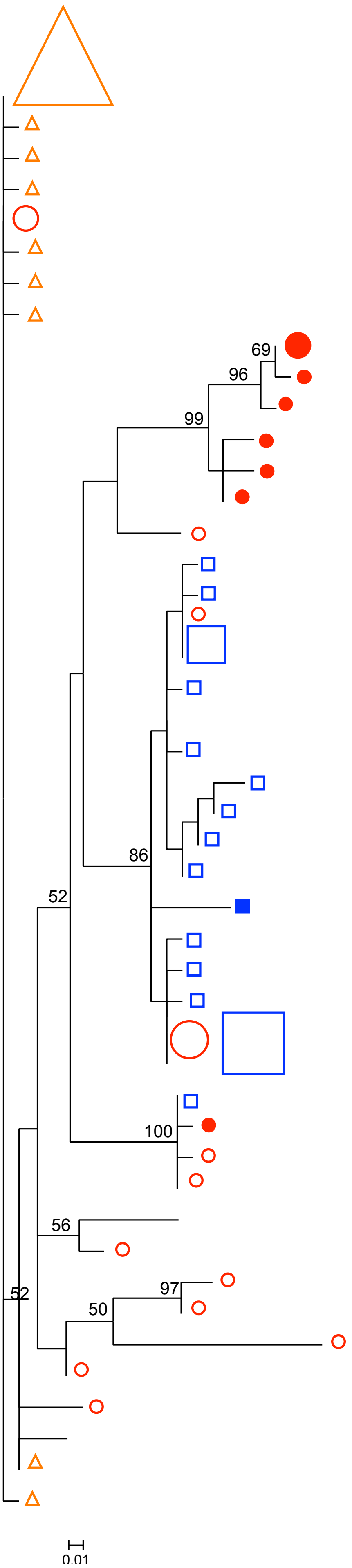

Subject 14

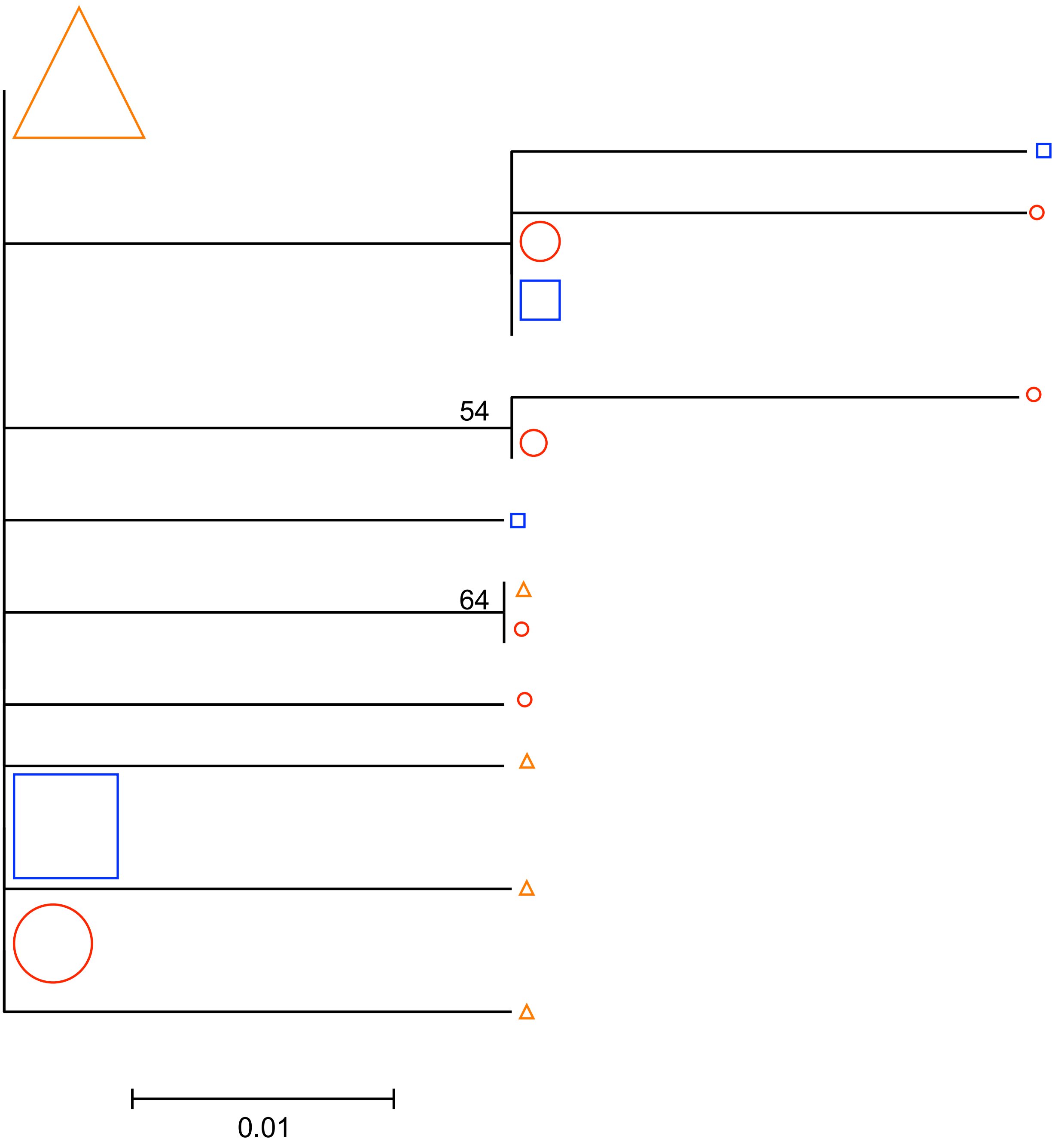

Subject 15

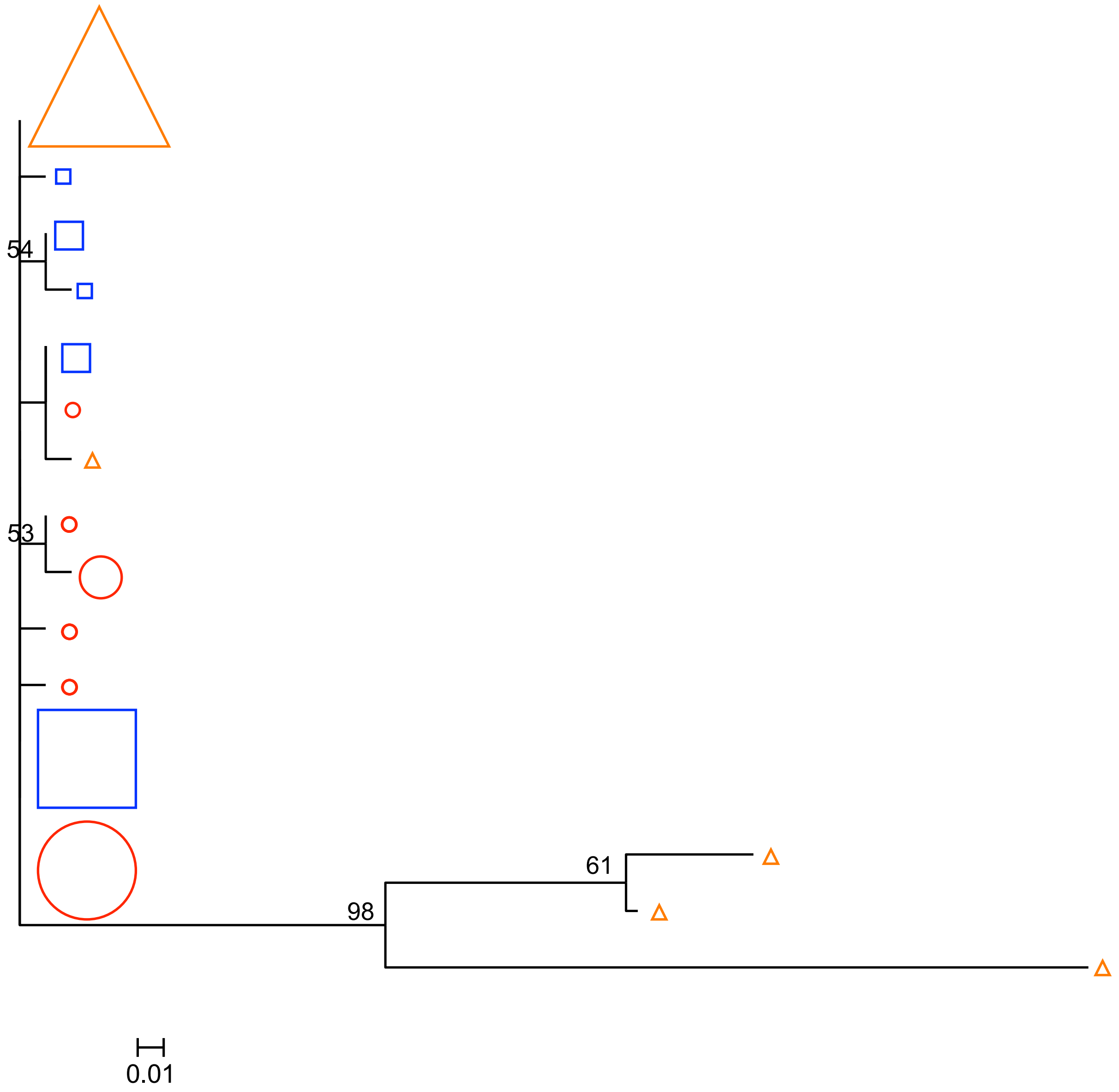

Subject 16

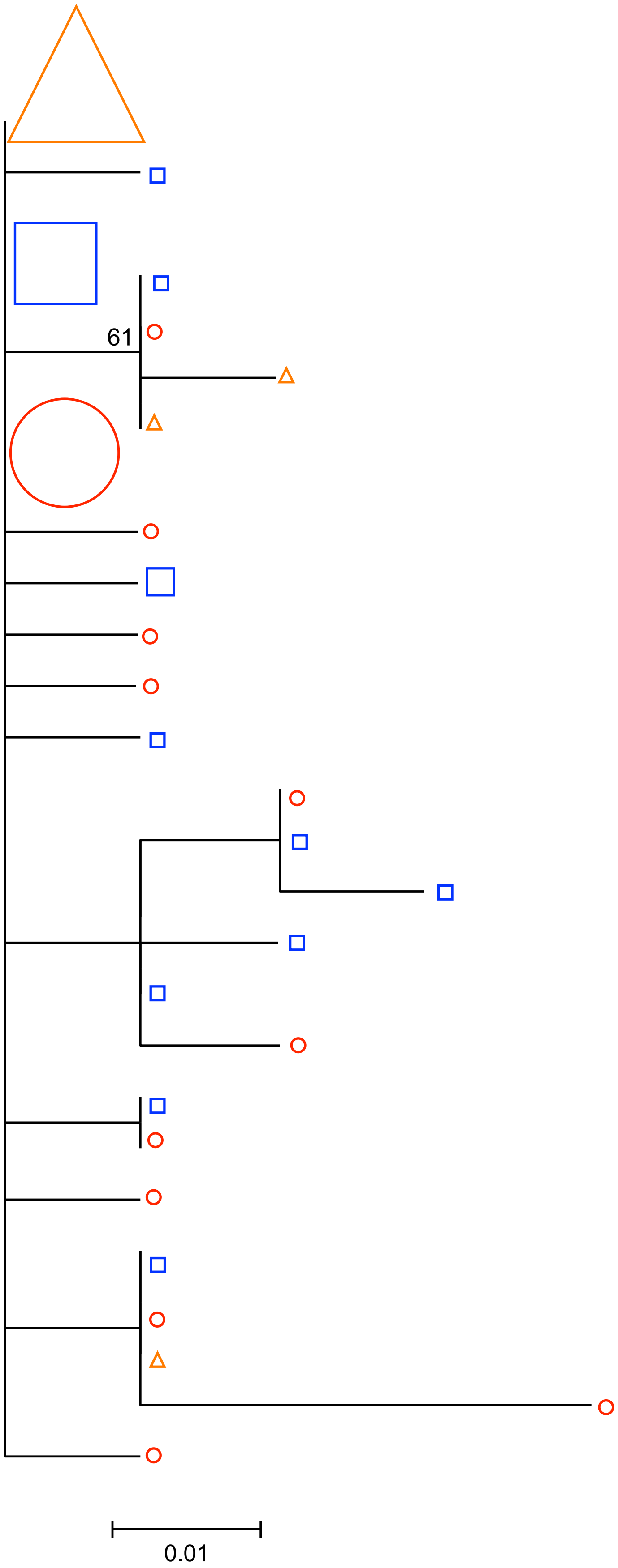

Subject 17

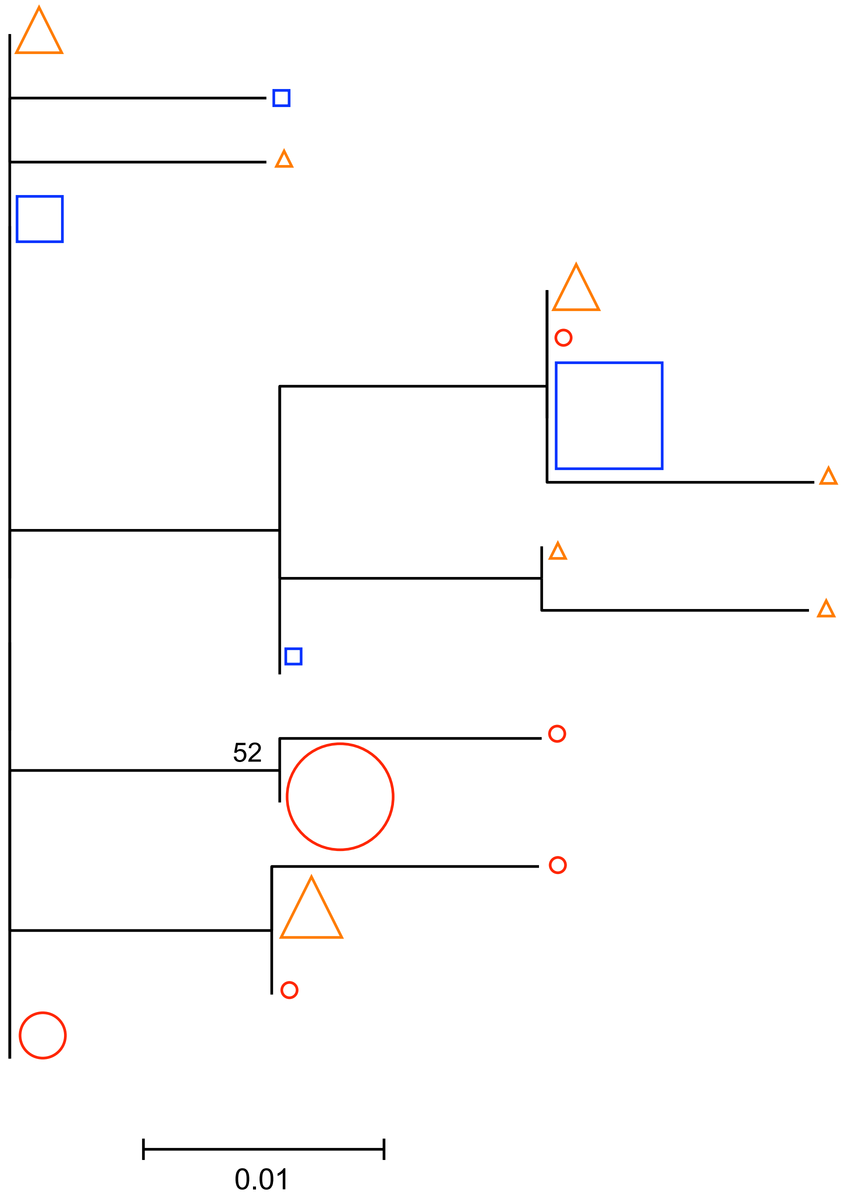

Subject 18

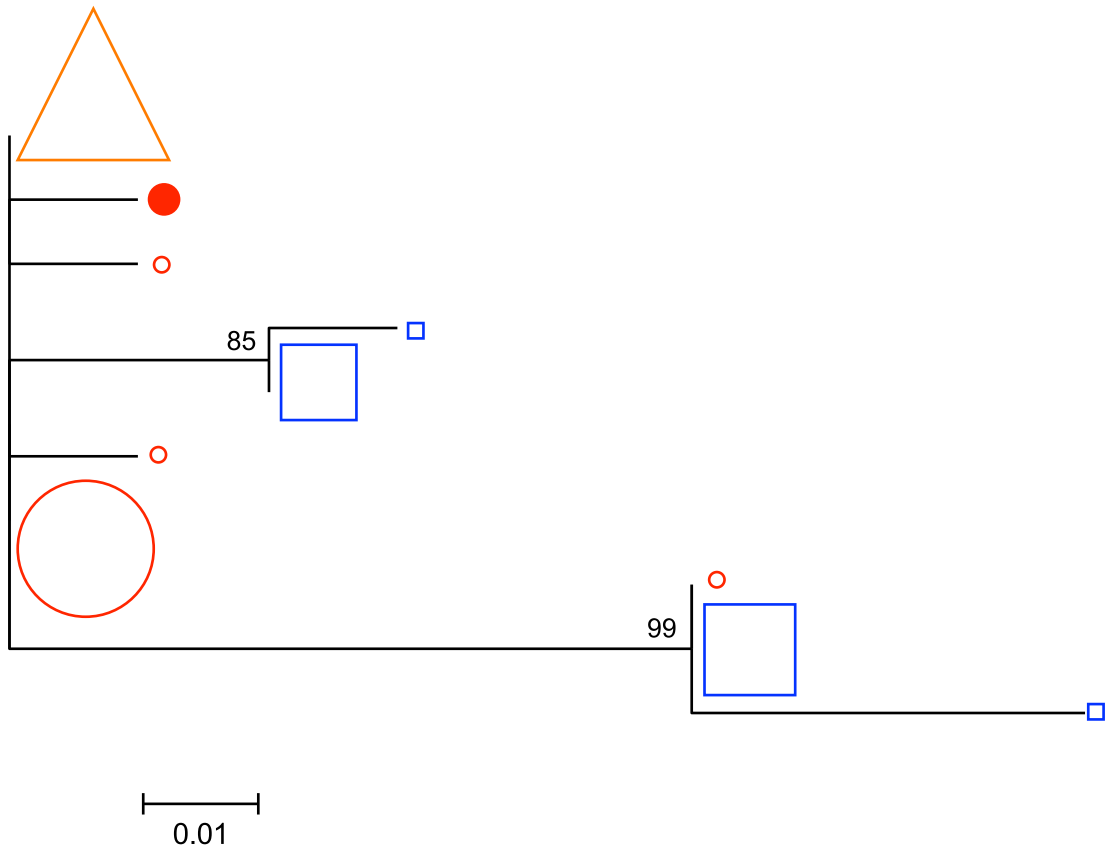

Subject 19

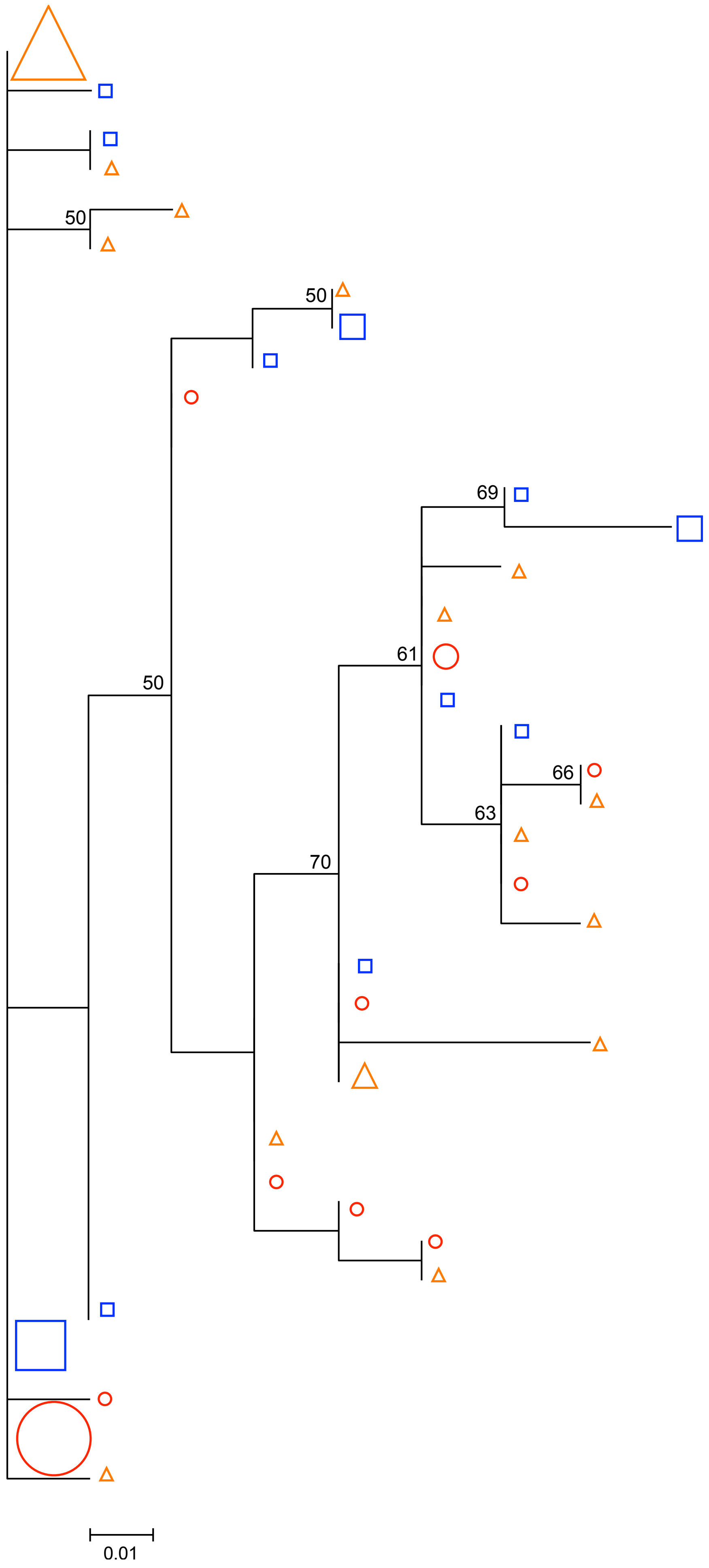

Subject 20

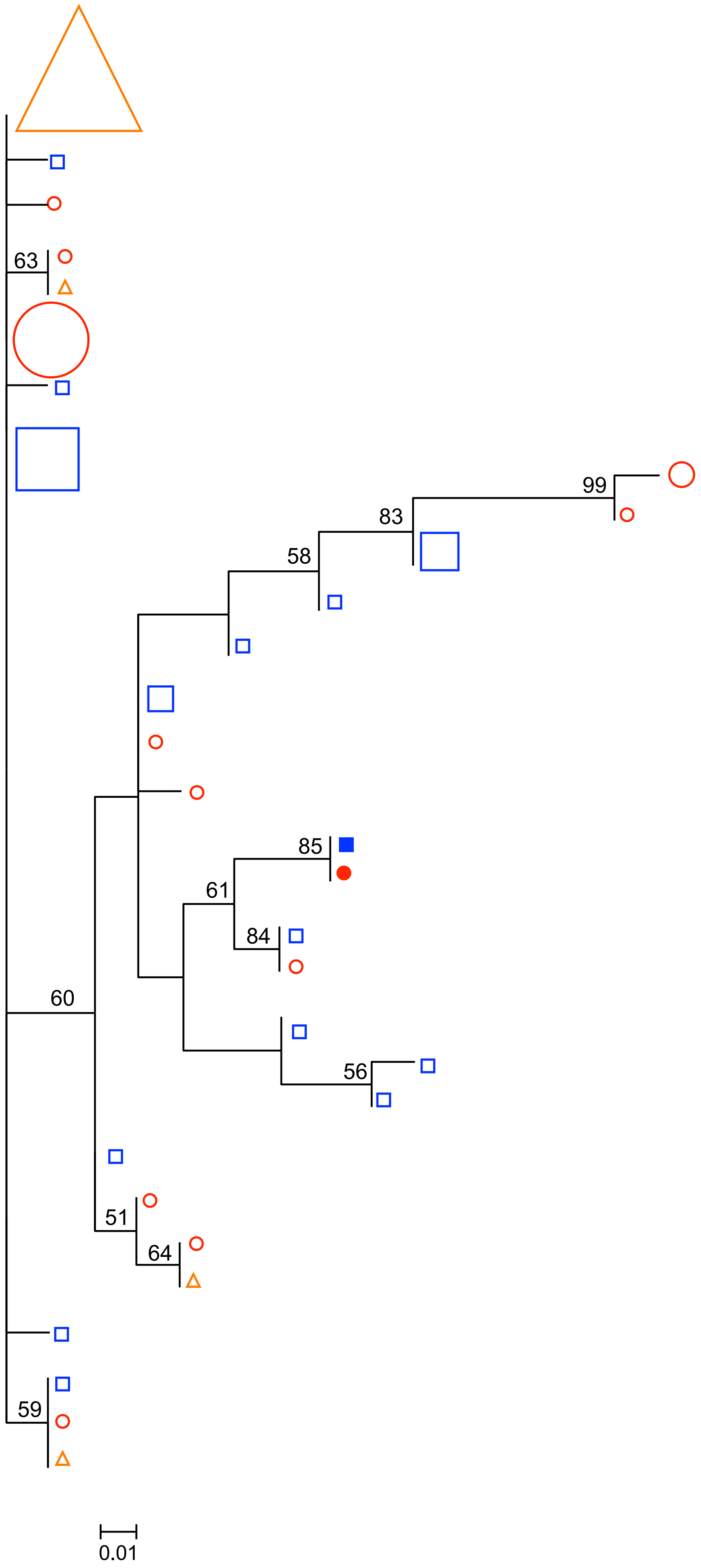

Subject 21

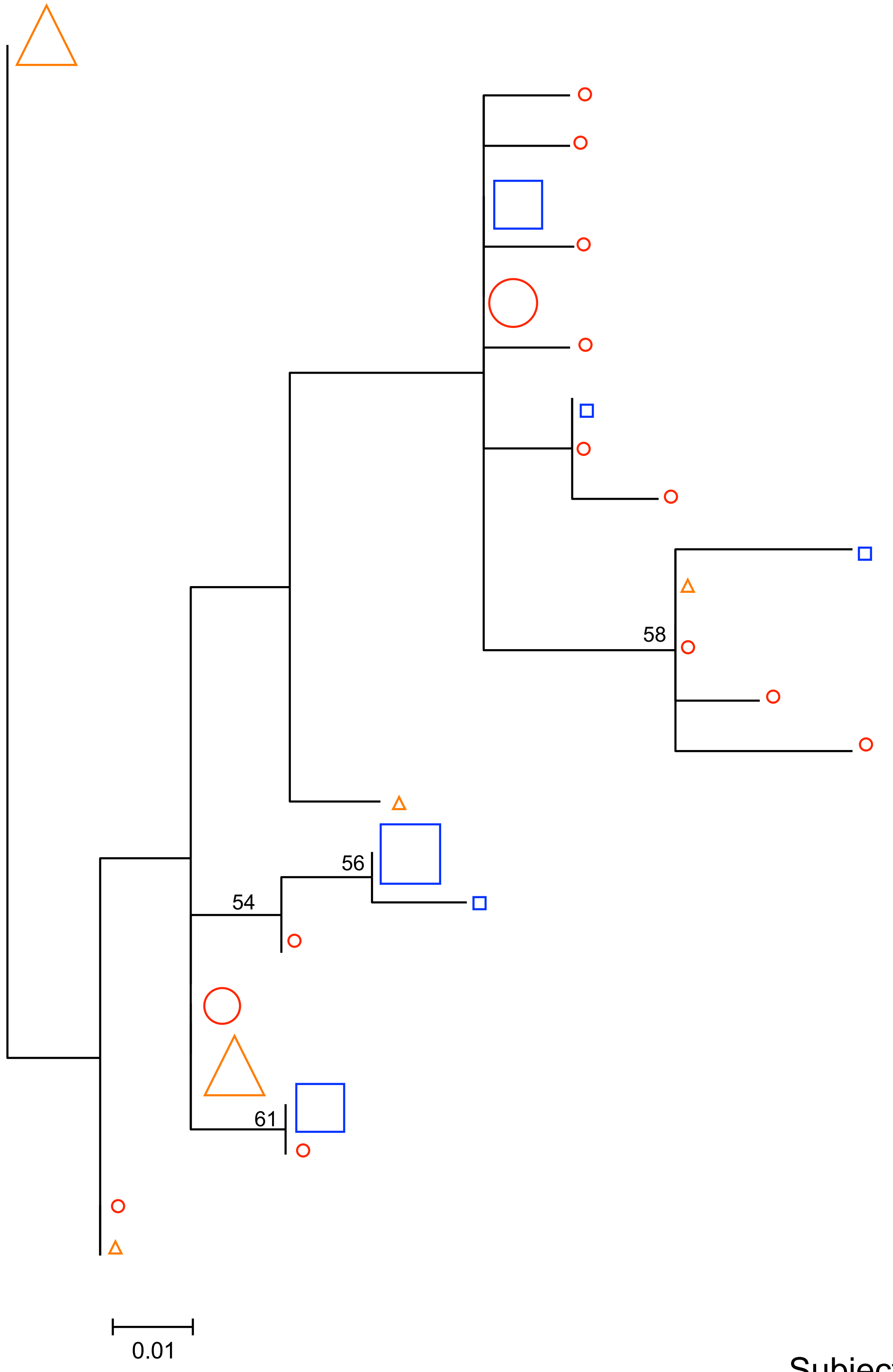

Subject 22

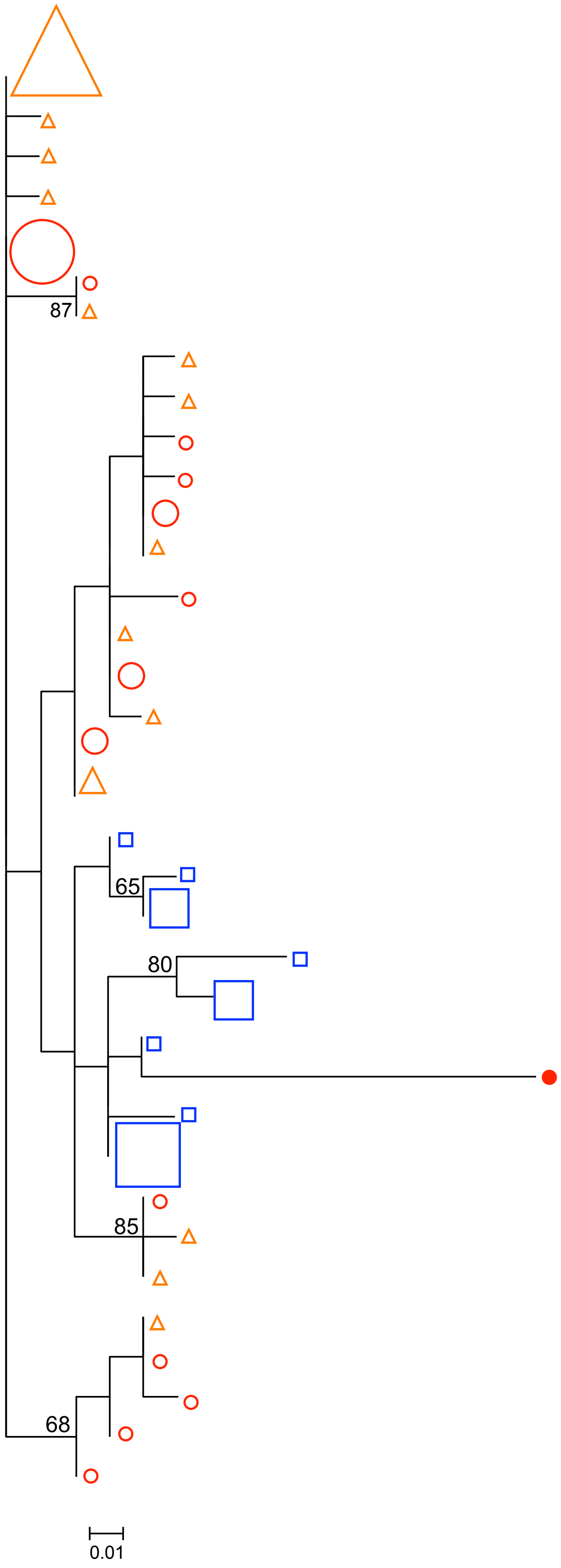

Subject 23

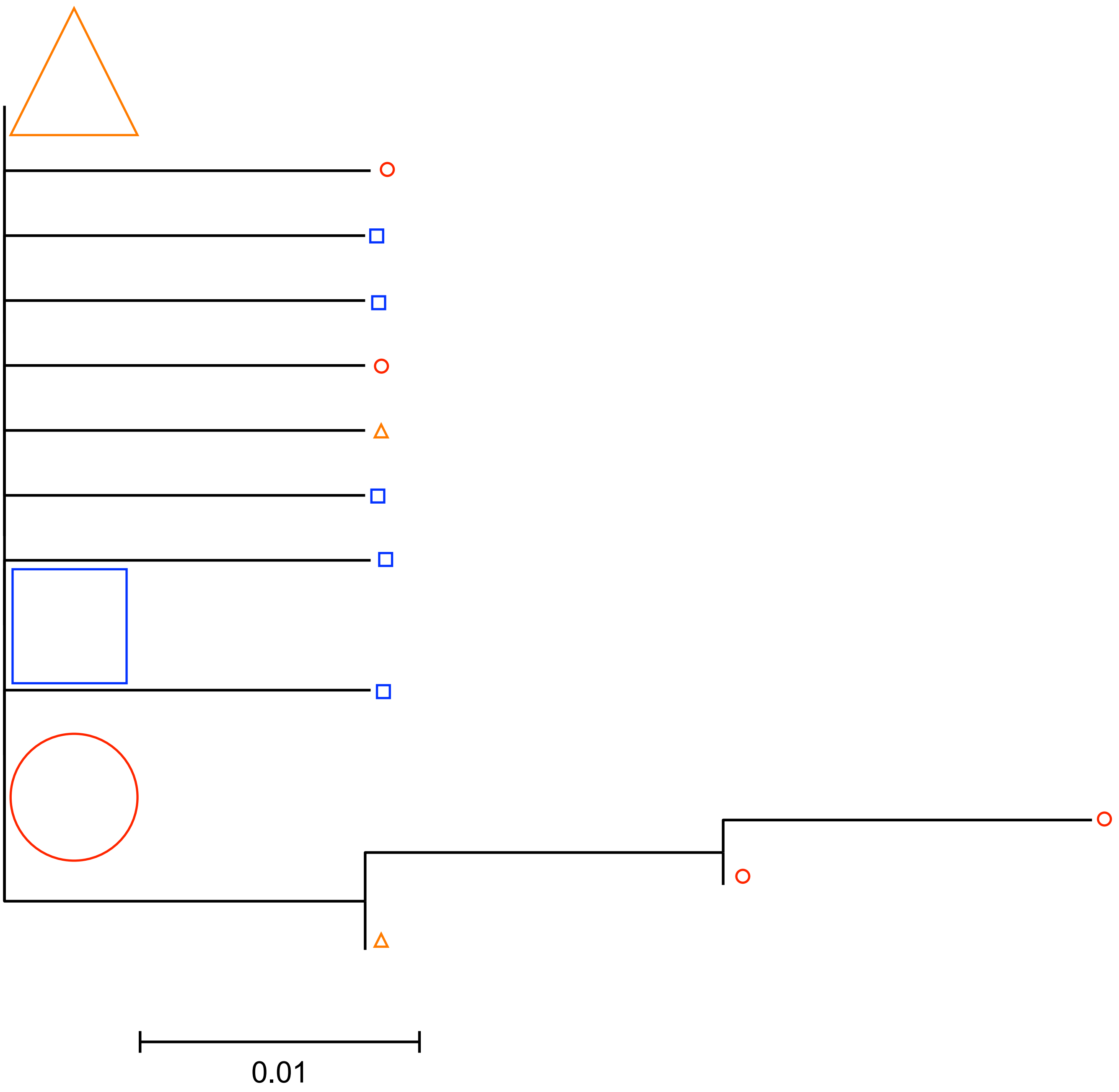

Subject 24

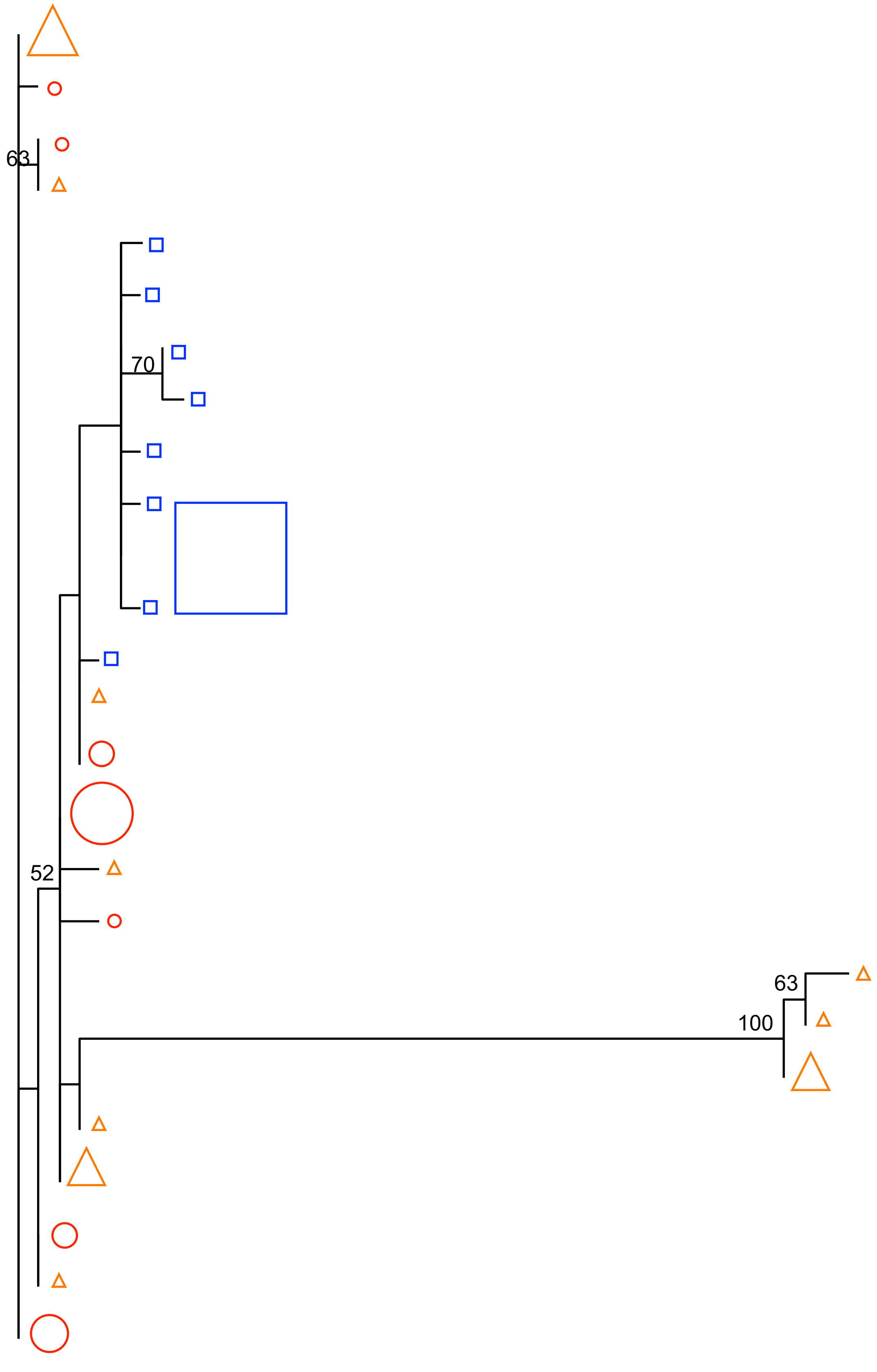

Subject 25

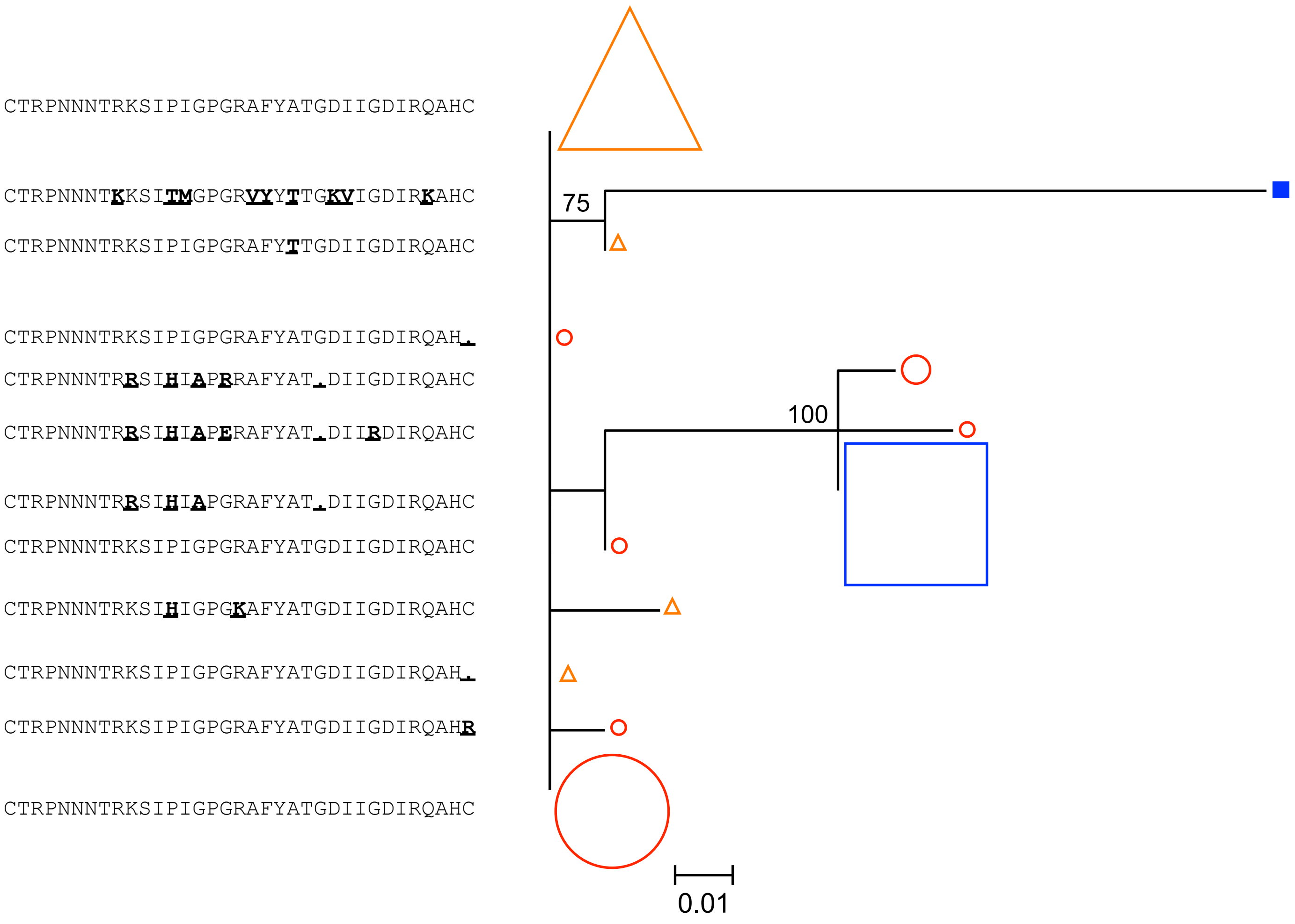
CTRPNNNTRKSIPIGPGRAFYATGDIIGDIRQAHC
CTRPNNNTKKSITMGPGRVYYTTGKVIGDIRKAHC
CTRPNNNTRKSIPIGPGRAFYTTGDIIGDIRQAHC
CTRPNNNTRKSIPIGPGRAFYATGDIIGDIRQAH.
CTRPNNNTRRSIHIAPRRAFYAT.DIIGDIRQAHC
CTRPNNNTRRSIHIAPERAFYAT.DIIRDIRQAHC
CTRPNNNTRRSIHIAPGRAFYAT.DIIGDIRQAHC
CTRPNNNTRKSIPIGPGRAFYATGDIIGDIRQAHC
CTRPNNNTRKSIHIGPGKAFYATGDIIGDIRQAHC
CTRPNNNTRKSIPIGPGRAFYATGDIIGDIRQAH.
CTRPNNNTRKSIPIGPGRAFYATGDIIGDIRQAHR
CTRPNNNTRKSIPIGPGRAFYATGDIIGDIRQAHC
75
100
0.01

Subject 26

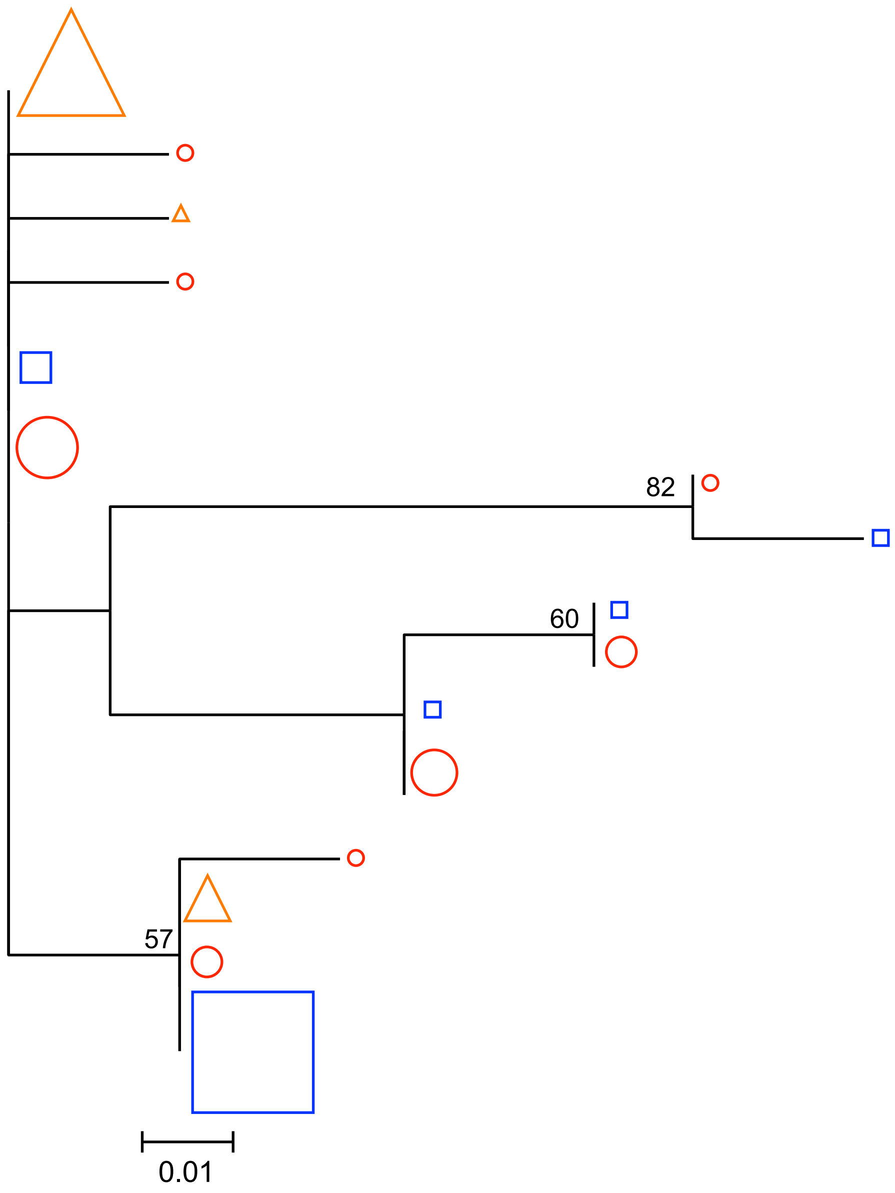

Subject 27

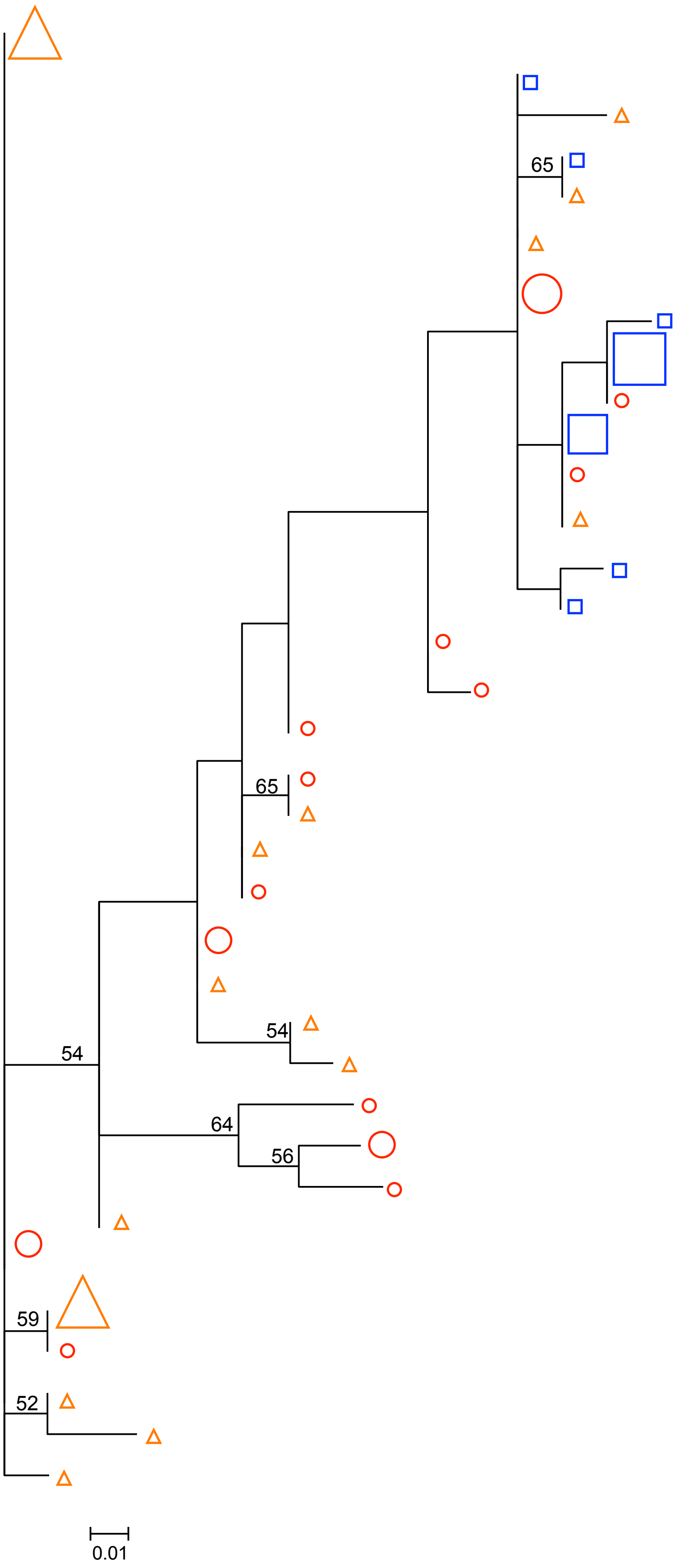

Subject 28

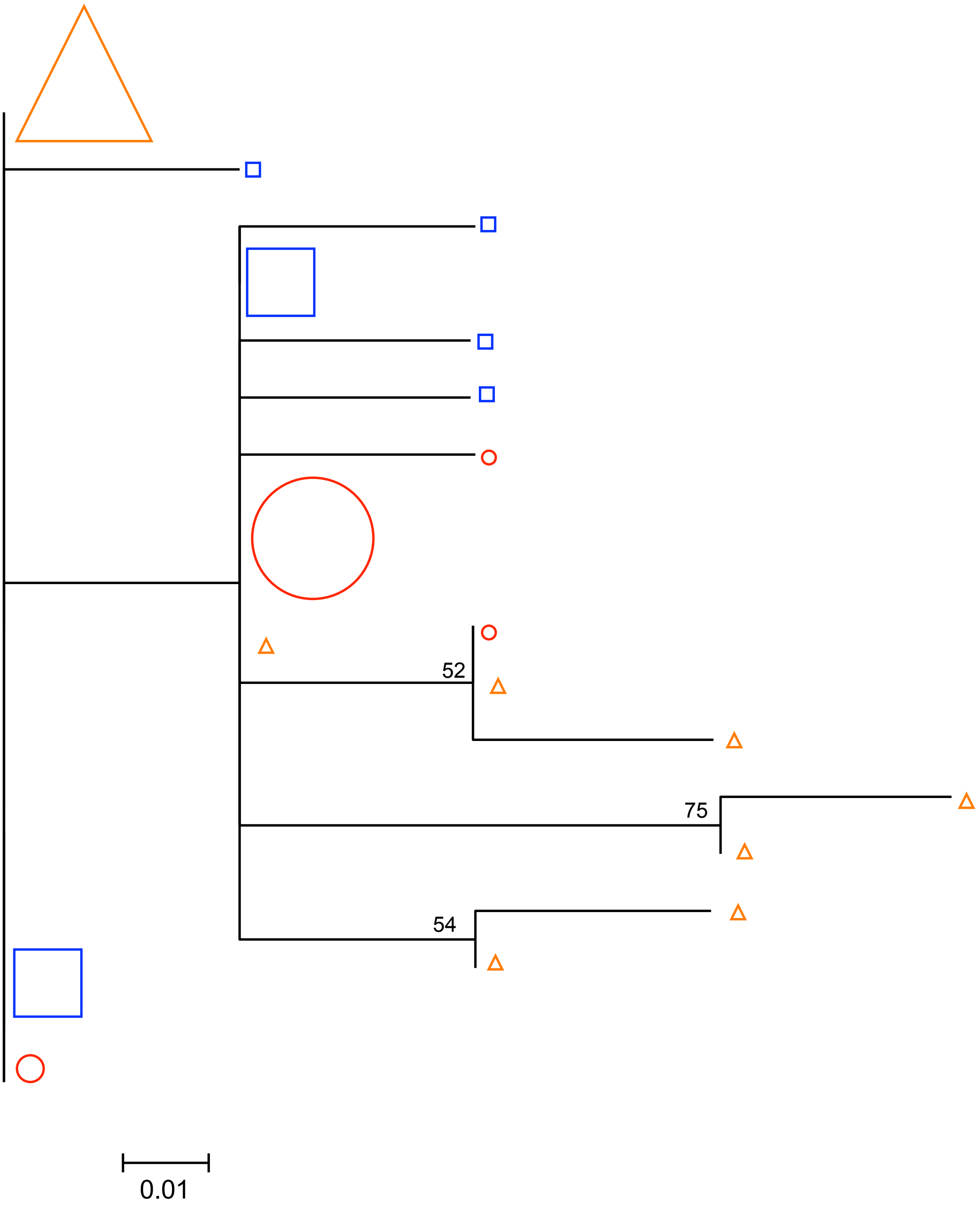

Subject 29

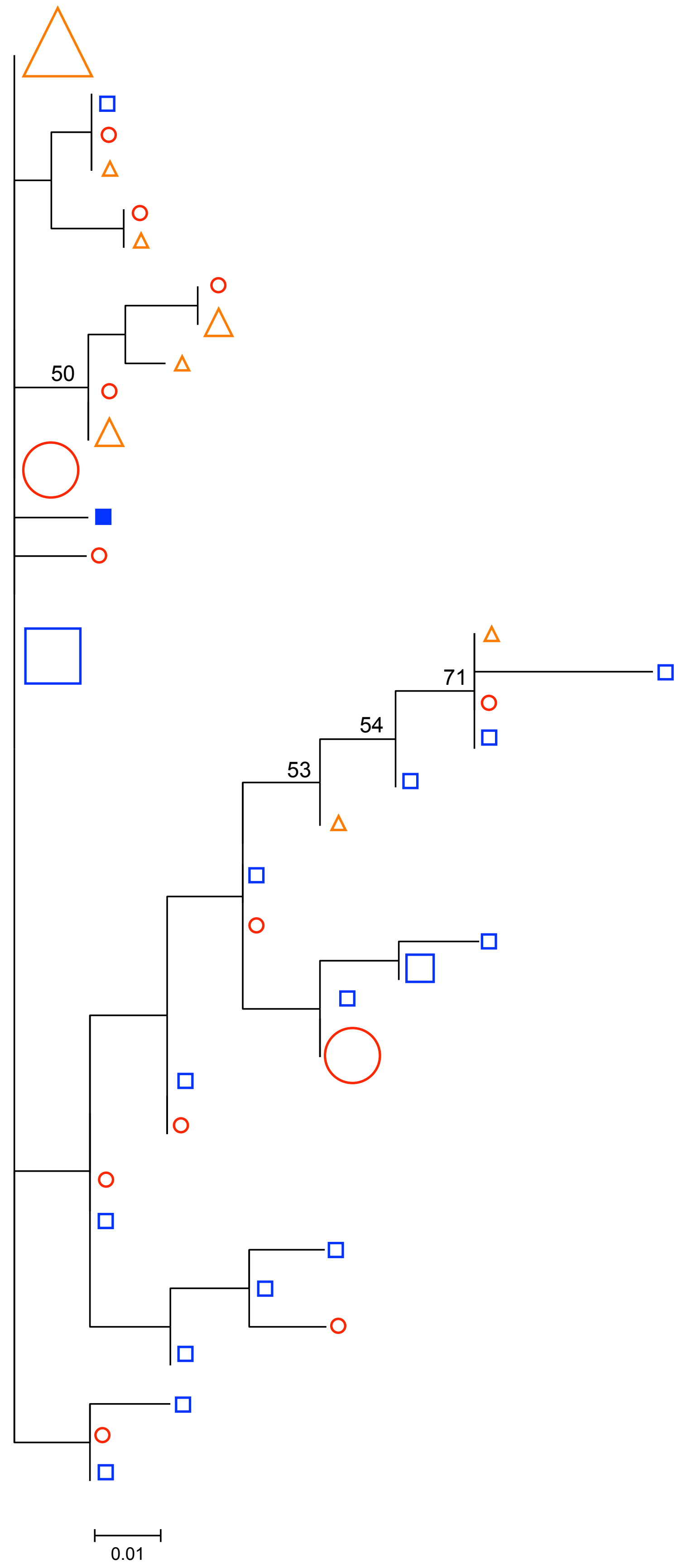

Subject 30
